# Machine learning prediction of ARDS after heart valve surgery: development and validation in Northwest China

**DOI:** 10.3389/fcvm.2025.1696326

**Published:** 2026-01-21

**Authors:** Xuhua Li, Hao Chen, Aoxiang Chen, Wenhao Zhan, Hengxi Zhang, Qiyuan Bai, Yalan Zhang, Bing Song

**Affiliations:** 1 The First Clinical Medical College of Lanzhou University, Lanzhou, China; 2 Mozi Laboratory, Zhengzhou, China; 3 School of Mechanical Engineering, University of Science and Technology Beijing, Beijing, China; 4 School of Computer Science and Technology, Xi'an Jiaotong University, Xi'an, China; 5 Department of Cardiovascular Surgery, The First Hospital of Lanzhou University, Lanzhou, China

**Keywords:** heart valve disease, extracorporeal circulation, acute respiratory distress syndrome (ARDS), risk factors, predictive model, machine learning

## Abstract

**Objective:**

To develop an AI-based predictive model for acute respiratory distress syndrome (ARDS) following cardiopulmonary bypass (CPB)-assisted heart valve replacement (HVR) to enable early identification of high-risk patients.

**Methods:**

We retrospectively analyzed 400 patients who underwent CPB-assisted HVR between January 2023 and February 2025. After data preprocessing and feature selection, the dataset was split into training (*n* = 280) and test (*n* = 120) sets. Multiple machine learning models were developed and optimized, with XGBoost emerging as the optimal model based on training performance.

**Results:**

Among 400 patients, 56 (14%) developed ARDS postoperatively. Key predictors included Age, absolute monocyte count,right atrial transverse diameter, intraoperative blood loss, platelet count, main pulmonary artery diameter. The XGBoost model achieved excellent performance with an AUC of 0.853 and demonstrated good calibration (HL test *p >* 0.05).

**Conclusion:**

The XGBoost model accurately predicts ARDS risk following CPB-assisted HVR using six clinically relevant predictors, providing a valuable tool for early risk stratification and potential intervention in high-risk patients.

## 1 Introduction

Over the past five decades, the global epidemiological profile of valvular heart disease (VHD) has shifted considerably. In high-income countries, degenerative valvular diseases have surpassed rheumatic heart disease as the most common etiology, while rheumatic forms continue to impose a major disease burden in developing nations. China, as a rapidly developing country undergoing epidemiological transition, currently faces a dual challenge from both rheumatic and degenerative VHD. According to the China Cardiovascular Health and Disease Report, the national weighted prevalence of VHD is 3.8%, affecting approximately 25 million people—a number expected to rise with the aging population (1). VHD impairs cardiac function and can lead to symptoms such as dyspnea, fatigue, and palpitations. Without intervention, serious complications including heart failure, arrhythmias, and thromboembolism may occur (2). Surgical treatments, such as valve repair or replacement, significantly improve hemodynamics, alleviate symptoms, and enhance quality of life. Advances in surgical techniques and perioperative management have substantially improved outcomes, even among elderly and high-risk patients, with operative mortality now below 5% in many settings (3). Although minimally invasive interventions have emerged as valuable alternatives, traditional open heart valve replacement remains the mainstay in cases of complex valvular lesions or in regions with limited access to advanced techniques (4, 5).

Open heart valve surgery relies fundamentally on CPB. This technology diverts venous blood via cannulae placed in the great veins or right atrium; the blood is then oxygenated and cleared of carbon dioxide through a membrane oxygenator, temperature-regulated via a heat exchanger, and filtered to remove microemboli before being returned to the systemic circulation through an arterial cannula (6, 7). *n* doing so, CPB completely supplants the functions of the heart and lungs, permitting a bloodless and motionless surgical field. Additionally, by inducing cardiac arrest under hypothermic conditions, CPB greatly reduces myocardial metabolic demand and facilitates precise operative repair (6). Since John Gibbon's first successful use of the heart-lung machine in 1953, CPB has become indispensable in cardiac surgery, enabling complex procedures and markedly improving operative safety and success (7, 8).

Nevertheless, CPB is a double-edged sword. Its use elicits a range of pathophysiological responses, including activation of the complement and coagulation systems and a systemic inflammatory response syndrome (SIRS), resulting from blood contact with non-endothelial surfaces (9). The lungs, receiving the entire cardiac output, are particularly vulnerable to SIRS and ischemia-reperfusion injury associated with CPB (10). These mechanisms can induce increased pulmonary vascular permeability, edema, hypoxemia, and pulmonary hypertension, culminating in acute lung injury (ALI)/ARDS. One study reported an ARDS incidence of 37.5% in infants following CPB surgery for congenital heart disease (11). Respiratory complications are among the leading causes of intensive care unit (ICU) readmission after cardiac surgery, accounting for 39% of cases (12, 13). Notably, CPB-associated ARDS carries a mortality rate of 50%–70% (14) and represents a major cause of postoperative death (14, 15). ARDS significantly increases 30-day mortality and hospital costs, and effective targeted treatments remain limited (16–18).

Thus, there is a pressing clinical need for early prediction tools to identify high-risk patients and facilitate timely interventions (18). This study analyzes data from patients who underwent CPB-assisted heart valve replacement at the Department of Cardiovascular Surgery, First Hospital of Lanzhou University, between January, 2023, and February, 2025. Using artificial intelligence methods, we aim to develop a risk prediction model for ARDS following valve surgery. This model may assist in early identification of high-risk individuals, supporting targeted preventive strategies to reduce the incidence of ARDS and improve patient outcomes.

## 2 Materials and methods

### 2.1 Study population and data collection

We conducted a retrospective analysis of consecutive patients who underwent CPB-assisted heart valve replacement surgery at the Department of Cardiovascular Surgery, First Hospital of Lanzhou University, from January 2023 to February 2025. A total of 400 patients who met the inclusion criteria were enrolled in this study.

Inclusion criteria: (1) Echocardiographically confirmed VHD; (2) Underwent heart valve replacement surgery with extracorporeal circulation assistance; (3) Age between 18 and 70 years.

Exclusion criteria: (1) History of prior cardiac surgery; (2) Inability to wean from cardiopulmonary bypass or early postoperative death precluding assessment of acute respiratory distress syndrome; (3) History of immune system disorders; (4) Active malignancy undergoing long-term chemotherapy or other treatments affecting laboratory test results; (5) Preoperative pulmonary comorbidities; (6) Patients with incomplete documentation; (7) Pregnancy.

Data collected included preoperative characteristics and comorbidities, intraoperative parameters (total CPB time, aortic cross-clamp time, blood loss, transfusion details, ultrafiltration volume, and urine output), and postoperative variables (blood gas analyses, inflammatory markers, and imaging findings). Ethical approval for this study was obtained from the Ethics Committee of the First Hospital of Lanzhou University (Approval No: LYDDLL2024-609). The requirement for informed consent was waived owing to the retrospective design of the research.

### 2.2 Outcome variable

The primary outcome was the occurrence of ARDS within 12 h after surgery. ARDS was defined according to adapted criteria from the American-European Consensus Conference (AECC, 1994) (19) and the Berlin Definition (2011) (20), as follows: (1) Acute onset of new or worsening respiratory symptoms within 1 week;(2) Bilateral pulmonary infiltrates on imaging not fully explained by effusions or atelectasis;(3) Non-cardiogenic pulmonary edema, assessed by pulmonary edema fluid/plasma protein ratio >0.6 or pulmonary artery wedge pressure ≤18 mmHg; (4) Impaired oxygenation: PaO₂/FiO₂ ≤ 300 mmHg with PEEP ≥5 cmH₂O.

### 2.3 Statistical analysis and predictive modeling

#### 2.3.1 Data preprocessing

All analyses were performed using Python (version 3.9.13). Categorical and continuous variables were expressed as frequencies (percentages) and mean *±* standard deviation, respectively. Features with >30% missing values were excluded. Exponential processing of cardiac chamber data and vascular diameters in echocardiography using the Doubais formula. Remaining missing data were imputed using Multiple Imputation by Chained Equations (MICE).Outliers were identified using the interquartile range (IQR) method, with outliers defined as values beyond *Q1–1.5IQR or Q3* *+* *1.5IQR* (21). Highly correlated features were identified through correlation heatmaps and clustering analyses and removed to avoid redundancy. Continuous and categorical variable distributions were visualized using kernel density estimation (KDE) plots and frequency bar charts, respectively. Associations among variables were examined using chi-square tests, *t*-tests, or non-parametric tests as appropriate. L1 regularization was incorporated in feature selection when significant correlations were observed.

#### 2.3.2 Data splitting and standardization

Continuous variables were standardized using StandardScaler. The dataset was randomly divided into training and testing sets at a 7:3 ratio. Group comparisons were performed using chi-square tests for categorical variables and t-tests or Mann–Whitney U tests for continuous variables.

#### 2.3.3 Feature selection and model development

Variable selection in this study is restricted to preoperative and intraoperative variables to develop a model capable of identifying high-risk patients before the conclusion of surgery.Feature selection was conducted using the Boruta algorithm and LASSO regression. To address class imbalance (36 ARDS cases, 12.86% in the training set), the SMOTEEN algorithm was applied to resample the training data. Eight machine learning models were evaluated: RandomForest, LightGBM, XGBoost, CatBoost, SVM, GBDT, ExtraTrees, and Logistic Regression. Model training and hyperparameter tuning employed 5-fold nested cross-validation and grid search, with the AUC serving as the primary metric for model selection.

#### 2.3.4 Model evaluation

The optimal model was validated on the test set. Performance was assessed using receiver operating characteristic (ROC) and precision-recall (PR) curves, along with sensitivity, specificity, accuracy, average precision, recall, and F1 score. Decision curve analysis (DCA) was performed to evaluate clinical utility. Model calibration was assessed using the Hosmer–Lemeshow (HL) test and calibration curves (CC). The SHAP (SHapley Additive exPlanations) framework was employed to interpret feature contributions and visualize results.

## 3 Results

### 3.1 Data processing

**Figure 1:**
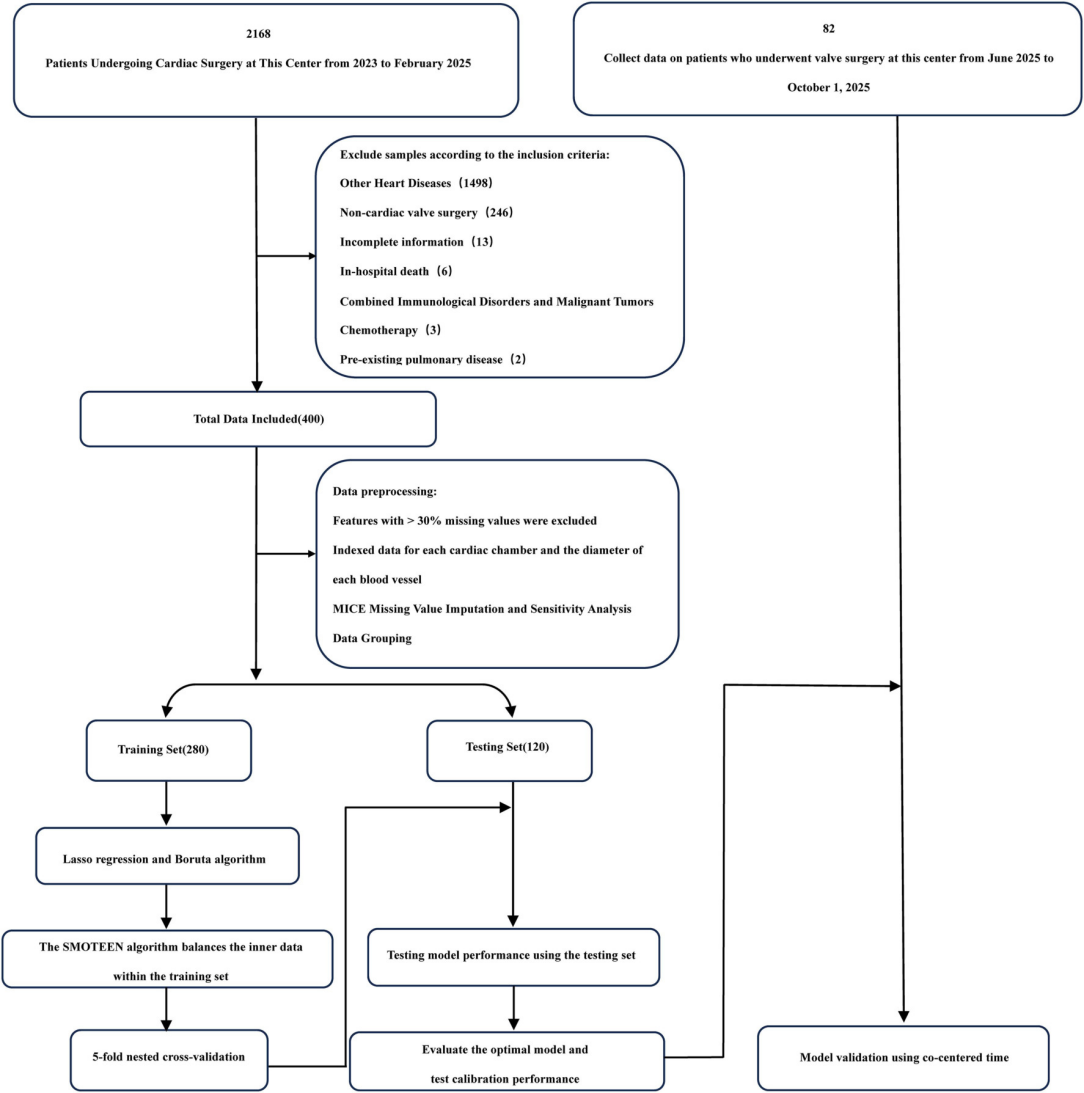
Flowchart.

**Figure 2:**
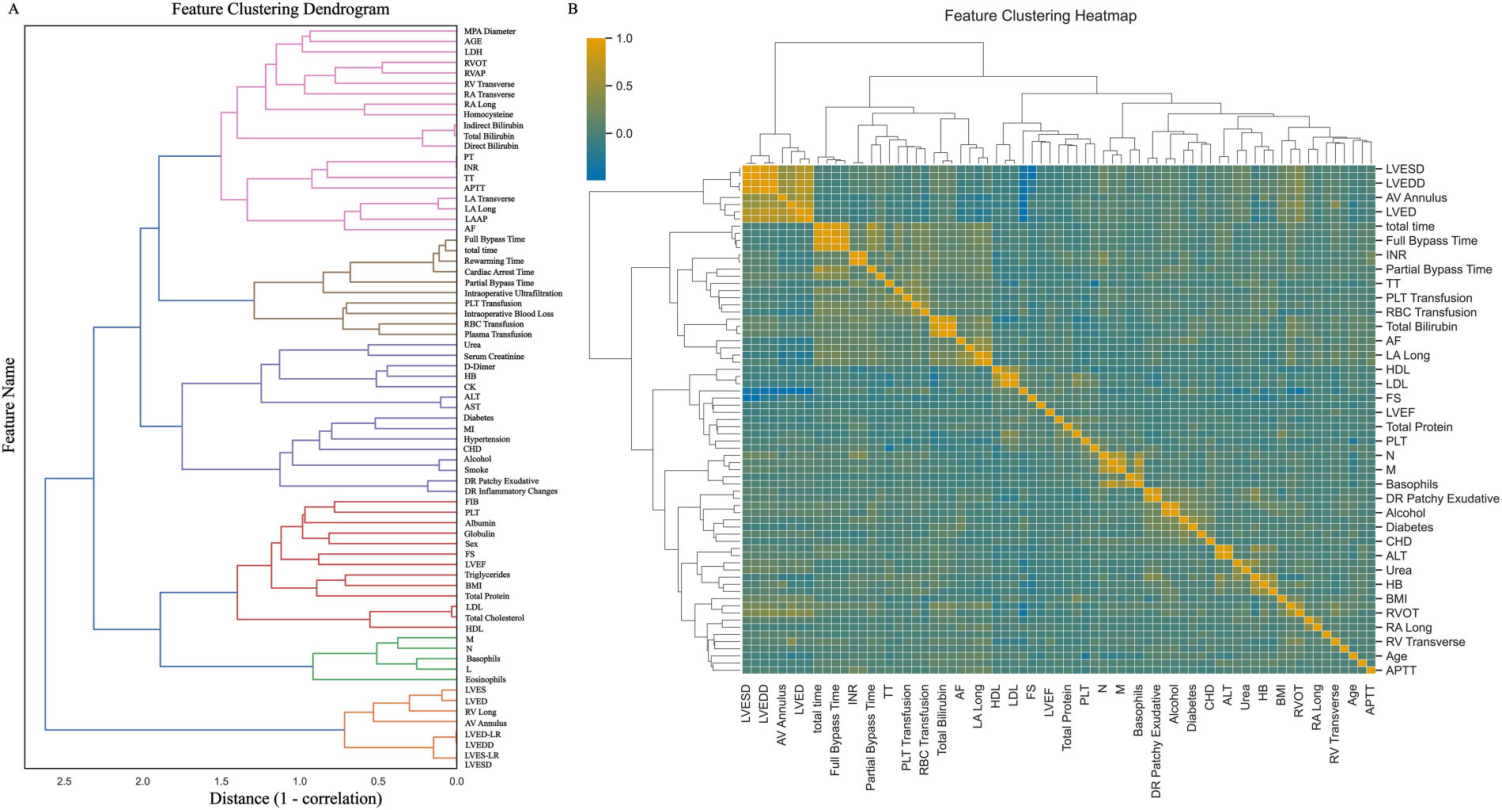
Feature clustering display. **(A)** Shows the feature clustering tree diagram; **(B)** Shows the feature clustering heat map. MPA Diameter: Main pulmonary artery diameter; LDH, lactate dehydrogenase; RVOT, right ventricular outflow tract; RVAP, right ventricular anteroposterior diameter; RV Transverse, right ventricular transverse diameter; RA Transverse, right atrial transverse dimension; RA Long, right atrial long axis; PT, prothrombin time; INR, international standardized ratio; TT, thrombin time; APTT, activated partial thromboplastin time; LA Transverse, left atrial transverse dimension; LA Long, left atrial long axis; LAAP, left atrial anterior-posterior diameter; AF, atrial fibrillation; total time, total duration of surgery; HB, hemoglobin; CK, creatine kinase; ALT, alanine aminotransferase; AST, aspartate aminotransferase; MI, myocardial infarction; CHD, coronary atherosclerotic disease; DR Patchy Exudative, x-ray chest radiograph showing patchy exudates; DR Inflammatory Changes, Inflammatory changes on chest x-ray; FIB, fibrinogen; PLT, platelet; FS, Left ventricular shortening fraction; LVEF, left ventricular ejection fraction; BMI, body mass index; LDL, low-density lipoprotein; HDL, how-density lipoprotein; M, absolute monocyte count; N, absolute neutrophil count; L, absolute lymphocyte count; LVES, left ventricular end systole; LVED, left ventricular end-diastolic length; RV Long, right ventricular long axis; AV Annulus, aortic annulus diameter; LVED-LR, left ventricular end-diastolic diameter; LVEDD, left ventricular end-diastolic diameter; LVES-LR, left ventricular end-systolic diameter; LVESD, left ventricular end-systolic anteroposterior diameter.

Among the 400 patient records included in the analysis, 56 (14.0%) developed ARDS during their ICU stay. No statistically significant differences were observed in baseline characteristics between groups (all *P* > 0.05). A comparison of baseline demographic and clinical data is summarized in Table 1, and a patient enrollment flowchart is provided in Figure 1. A heatmap illustrating missing data patterns across all variables is shown in Supplementary Figure S1, and the proportion of missing values per variable is presented in Supplementary Figure S2. The distribution of data after handling missing values is visualized in Supplementary Figure S3. Categorical and continuous variable distributions are displayed using bar charts (Supplementary Figure S4) and box plots (Supplementary Figure S5), respectively. No significant outliers were detected during data verification. Feature clustering analysis is illustrated in Figure 2. Sensitivity analysis following multiple imputation confirmed the robustness of the imputed dataset, as shown in Supplementary Figures 6, 7. Results from independence tests for categorical variables and correlation analyses for continuous variables are presented as heatmaps in Supplementary Figure 8.

**Table 1:** Baseline characteristics.

Variables	Non- ARDS (*n* = 344)	ARDS (*n* = 56)	*p*-value
Men	53.50%	66.10%	0.11
Alcohol	7.80%	7.10%	1.00
Smoke	9.60%	8.90%	1.00
Hypertension	23.00%	14.30%	0.20
CHD	31.10%	41.10%	0.19
MI	2.30%	0.00%	0.61
AF	23.90%	28.6%	0.56
Diabetes	5.80%	3.60%	0.75
Aortic valve regurgitation degree			0.69
Mild	14.50%	20.00%	0.54
Mild to Moderate	10.70%	5.00%	0.43
Moderate	16.40%	12.50%	0.72
Moderate to Severe	13.80%	12.50%	1.00
Severe	44.70%	50.00%	0.67
Mitral valve regurgitation degree			0.62
Mild	28.60%	31.1%	0.92
Mild to Moderate	2.40%	8.7%	0.31
Moderate	23.80%	19.4%	0.71
Moderate to Severe	11.90%	7.8%	0.64
Severe	33.30%	33.0%	1.00
Tricuspid valve regurgitation degree			0.08
Mild	18.20%	0.00%	0.26
Mild to Moderate	0.00%	8.30%	0.48
Moderate	9.10%	8.30%	1.00
Moderate to Severe	2.30%	0.00%	1.00
Severe	2.30%	16.70%	0.22
Age	52.69 ± 11.06	55.59 ± 8.50	0.09
Height	165.45 ± 8.61	167.79 ± 8.09	0.07
Weight	63.34 ± 10.99	65.92 ± 12.05	0.14
BMI	23.06 ± 3.14	23.32 ± 3.35	0.57
HB	142.75 ± 22.82	155.43 ± 113.38	0.33
WBC	5.63 ± 2.09	5.81 ± 2.29	0.53
PLT	185.83 ± 59.30	177.56 ± 62.11	0.30
RBC	4.71 ± 0.54	4.72 ± 0.71	0.86
Total Bilirubin	18.58 ± 9.99	18.44 ± 7.51	0.90
Direct Bilirubin	3.74 ± 2.38	3.93 ± 2.07	0.49
Indirect Bilirubin	14.79 ± 8.04	14.52 ± 5.95	0.75
Total Protein	74.98 ± 69.33	67.98 ± 6.23	0.11
Albumin	44.24 ± 21.57	40.83 ± 4.55	<0.05
Globulin	27.15 ± 4.94	26.82 ± 4.42	0.60
Serum Creatinine	74.63 ± 28.82	76.67 ± 35.54	0.65
Urea	6.98 ± 4.72	6.76 ± 2.28	0.57
Total Cholesterol	4.02 ± 1.01	3.96 ± 0.98	0.66
Triglycerides	1.45 ± 0.98	1.33 ± 0.64	0.19
BNP	1,289.37 ± 2,973.09	1,628.70 ± 2,574.09	0.50
INR	1.20 ± 0.61	1.21 ± 0.44	0.84
APTT	34.62 ± 19.32	33.91 ± 3.90	0.57
LVEF	58.76 ± 30.80	55.54 ± 6.92	0.11

CHD, coronary atherosclerotic heart disease; MI, myocardial infarction; AF, atrial fibrillation; BMI, body mass index; HB, hemoglobin; WBC, white blood cell; PLT, platelet; RBC, red blood cell; BNP, B-type brain natriuretic peptide; INR, international normalized ratio; APTT, activated partial thromboplastin time; LVEF, left ventricular ejection fraction.

### 3.2 Feature selection

**Figure 3:**
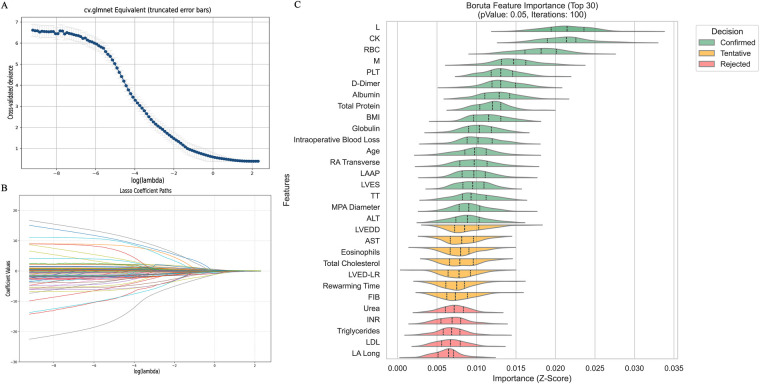
Feature selection diagram. **(A)** Shows the cross-validation plot of the Lasso regression algorithm; **(B)** shows the path plot of the Lasso regression coefficients; **(C)** Shows the feature selection plot of the Boruta algorithm. L, absolute lymphocyte count; CK, creatine kinase; M, absolute monocyte count; BMI, body mass index; RA Transverse, right atrial transverse dimension; LAAP, left atrial anterior-posterior diameter; LVES, left ventricular end systole; TT, thrombin time; MPA Diameter, main pulmonary artery diameter; ALT, alanine aminotransferase; LVEDD, left ventricular end-diastolic diameter; AST, aspartate aminotransferase; LVED-LR, eft ventricular end-diastolic diameter; FIB, fibrinogen; INR, international standardized ratio; LDL, low-density lipoprotein; LA Long, left atrial long axis.

**Figure 4:**
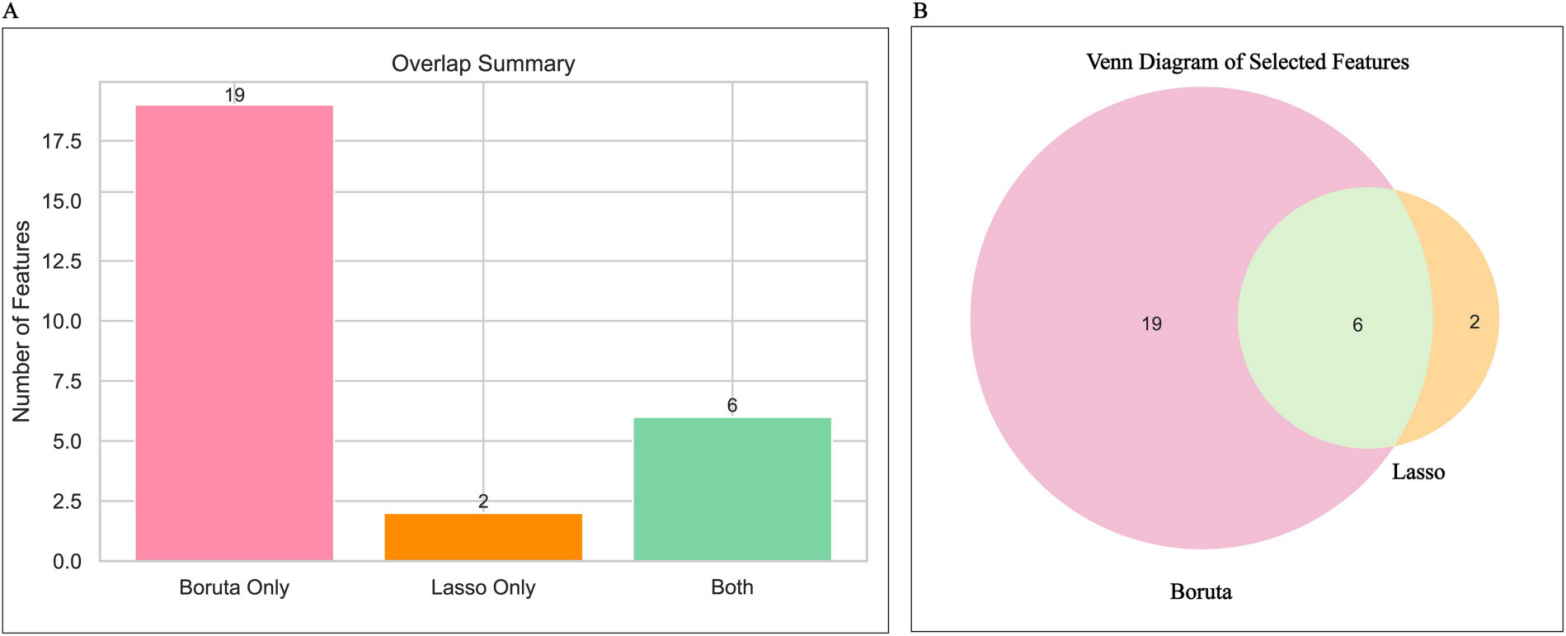
Feature determination display. **(A)** Shows a summary of the number of features for Lasso regression and the Boruta algorithm; **(B)** Shows the Venn diagram of features selected by Lasso regression and the Boruta algorithm.

After dataset splitting, categorical and continuous variables were assessed using chi-square and t-tests, respectively, to evaluate the balance between training and testing sets. Most variables showed no significant differences (*P* > 0.05), with the exception of intraoperative blood loss (*P* < 0.05). Detailed test results are provided in Table 2. Given the observed associations and correlations among features—as indicated by clustering, independence testing, and Pearson correlation heatmaps—feature selection was performed using both Lasso regression (with L1 regularization; Figures 3A,B) and the Boruta algorithm (Figure 3C). The intersection of features identified by both methods was retained (Figure 4). The final selected features were: Age, absolute monocyte count, right atrial transverse diameter, intraoperative blood loss, platelet count, main pulmonary artery diameter.

**Table 2:** Dataset partitioning verification.

Variables	Train set	Test set	*p-*value
	(%/*X* ± *SD*)	(%/*X* ± *SD*)	
Women	44.60%	45.00%	1.00
Smoke	8.60%	11.70%	0.35
Alcohol	6.80%	10.00%	0.31
Hypertension	23.20%	18.30%	0.29
CHD	34.30%	26.70%	0.49
MI	2.90%	0.00%	0.14
AF	24.60%	24.20%	0.80
Diabetes	4.60%	7.50%	0.42
DR Inflammatory Changes	10.00%	5.80%	0.25
DR Patchy Exudative	11.40%	5.00%	0.06
Age	0.01 ± 1.03	−0.03 ± 0.93	0.68
BMI	−0.02 ± 1.01	0.04 ± 0.99	0.56
HB	−0.05 ± 0.43	0.11 ± 1.71	0.30
WBC	−0.04 ± 1.04	0.10 ± 0.90	0.17
PLT	−0.03 ± 1.00	0.08 ± 1.01	0.32
RBC	−0.03 ± 0.99	0.07 ± 1.03	0.38
N	−0.00 ± 1.01	0.01 ± 0.97	0.92
L	0.01 ± 1.08	−0.02 ± 0.79	0.71
M	0.01 ± 1.10	−0.03 ± 0.72	0.61
Eosinophils	0.01 ± 1.13	−0.02 ± 0.59	0.76
Basophils	−0.02 ± 1.00	0.05 ± 1.01	0.50
AST	−0.05 ± 0.50	0.12 ± 1.66	0.29
ALT	−0.05 ± 0.45	0.12 ± 1.69	0.28
Total Bilirubin	−0.03 ± 0.92	0.08 ± 1.17	0.36
Direct Bilirubin	−0.02 ± 1.00	0.05 ± 1.02	0.51
Indirect Bilirubin	−0.03 ± 0.89	0.07 ± 1.22	0.41
Total Protein	−0.01 ± 0.98	0.03 ± 1.05	0.71
Globulin	0.02 ± 1.11	−0.04 ± 0.70	0.52
Albumin	0.02 ± 1.19	−0.06 ± 0.22	0.27
Serum Creatinine	−0.03 ± 0.81	0.07 ± 1.35	0.43
Urea	−0.01 ± 1.04	0.03 ± 0.92	0.68
Total Cholesterol	0.01 ± 1.01	−0.01 ± 0.98	0.87
Triglycerides	−0.00 ± 1.08	0.00 ± 0.80	0.98
HDL	−0.00 ± 1.03	0.01 ± 0.94	0.88
LDL	0.00 ± 1.00	−0.00 ± 1.00	0.95
LDH	−0.06 ± 0.33	0.13 ± 1.76	0.25
Homocysteine	−0.08 ± 0.58	0.18 ± 1.59	0.08
CK	−0.05 ± 0.72	0.13 ± 1.45	0.19
INR	0.03 ± 1.14	−0.07 ± 0.57	0.27
PT	0.03 ± 1.14	−0.07 ± 0.53	0.24
APTT	−0.04 ± 0.33	0.09 ± 1.76	0.44
D-Dimer	−0.03 ± 0.85	0.07 ± 1.28	0.46
FIB	0.04 ± 1.00	−0.09 ± 1.01	0.23
TT	−0.00 ± 1.13	0.00 ± 0.60	0.95
AV Annulus	−0.02 ± 0.99	0.04 ± 1.03	0.59
LAAP	0.02 ± 1.13	−0.05 ± 0.62	0.40
RVAP	0.00 ± 1.01	−0.01 ± 0.99	0.94
RVOT	0.00 ± 0.99	−0.00 ± 1.03	0.99
MPA Diameter	0.04 ± 1.12	−0.02 ± 0.24	0.28
LVEDD	0.01 ± 0.98	−0.01 ± 1.05	0.85
LVESD	0.01 ± 0.96	−0.01 ± 1.09	0.86
LVED-LR	0.00 ± 0.99	−0.01 ± 1.04	0.94
LVES-LR	0.00 ± 0.96	−0.01 ± 1.10	0.89
LVED	−0.02 ± 0.99	0.04 ± 1.04	0.65
LVES	−0.03 ± 0.96	0.07 ± 1.09	0.40
RV Long	0.02 ± 1.02	−0.05 ± 0.96	0.53
RV Transverse	0.03 ± 1.04	−0.08 ± 0.91	0.28
LA Long	0.02 ± 1.03	−0.05 ± 0.92	0.49
LA Transverse	0.00 ± 1.03	−0.00 ± 0.93	0.97
RA Long	−0.07 ± 0.19	0.17 ± 1.80	0.15
RA Transverse	0.04 ± 1.19	−0.10 ± 0.23	0.06
LVEF	0.03 ± 1.18	−0.08 ± 0.25	0.15
FS	−0.04 ± 0.89	0.09 ± 1.22	0.31
Total time	0.05 ± 1.01	−0.12 ± 0.96	0.11
Full Bypass Time	0.05 ± 1.00	−0.12 ± 1.01	0.11
Partial Bypass Time	0.05 ± 1.06	−0.11 ± 0.84	0.11
Cardiac Arrest Time	0.05 ± 1.02	−0.12 ± 0.96	0.10
Rewarming Time	0.04 ± 1.00	−0.10 ± 1.00	0.18
Intraoperative Blood Loss	0.08 ± 1.11	−0.18 ± 0.66	0.03
Plasma Transfusion	0.02 ± 1.00	−0.04 ± 1.02	0.63
RBC Transfusion	0.01 ± 1.03	−0.02 ± 0.94	0.83
PLT Transfusion	0.03 ± 1.05	−0.07 ± 0.87	0.33
Intraoperative Ultrafiltration	0.05 ± 1.06	−0.11 ± 0.86	0.12

MPA Diameter, main pulmonary artery diameter; LDH, lactate dehydrogenase; RVOT, right ventricular outflow tract; RVAP, right ventricular anteroposterior diameter; RV Transverse, right ventricular transverse diameter; RA Transverse, right atrial transverse dimension; RA Long, right atrial long axis; PT, prothrombin time; INR, international standardized ratio; TT, thrombin time; APTT, activated partial thromboplastin time; LA Transverse, left atrial transverse dimension; LA Long, left atrial long axis; LAAP, left atrial anterior-posterior diameter; AF, atrial fibrillation; total time, total duration of surgery; HB, hemoglobin; CK, creatine kinase; ALT, alanine aminotransferase; AST, aspartate aminotransferase; MI, myocardial infarction; CHD, coronary atherosclerotic disease; DR Patchy Exudative, x-ray chest radiograph showing patchy exudates; DR Inflammatory Changes, inflammatory changes on chest x-ray; FIB, fibrinogen; PLT, platelet; FS, left ventricular shortening fraction; LVEF, left ventricular ejection fraction; BMI, body mass index; LDL, low-density lipoprotein; HDL, how-density lipoprotein; M, absolute monocyte count; N, absolute neutrophil count; L, absolute lymphocyte count; LVES, left ventricular end systole; LVED, left ventricular end-diastolic length; RV Long, right ventricular long axis; AV Annulus, aortic annulus diameter; LVED-LR, left ventricular end-diastolic diameter; LVEDD, left ventricular end-diastolic diameter; LVES-LR, left ventricular end-systolic diameter; LVESD, left ventricular end-systolic anteroposterior diameter.

### 3.3 Model construction

**Figure 5:**
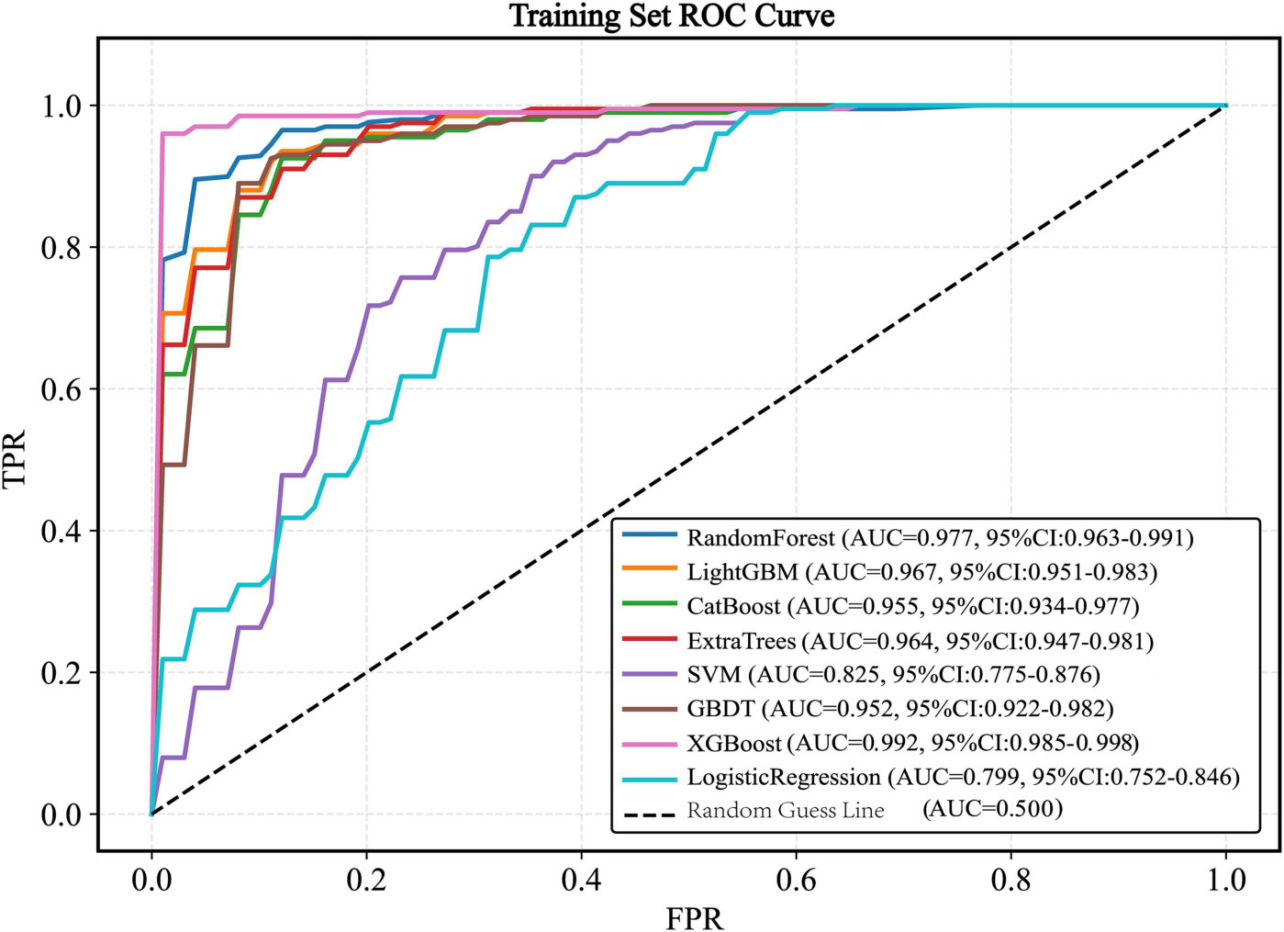
ROC curve of the train set. ROC, receiver operating characteristic; AUC, the area under the receiver operating characteristic curve; LightGBM, light gradient boosting machine; XGBoost, extreme gradient boosting; CatBoost, categorical boosting; SVM, support vector machine; GBDT, gradient boosting decision tree.

Eight machine learning models were trained on the balanced training set: Random Forest, LightGBM, XGBoost, CatBoost, SVM, GBDT, ExtraTrees, and Logistic Regression. Based on ROC-AUC evaluation within the training set, the XGBoost model demonstrated the highest performance (AUC = 0.992, 95% *CI* 0.985–0.998; Figure 5).

### 3.4 Model validation

**Figure 6:**
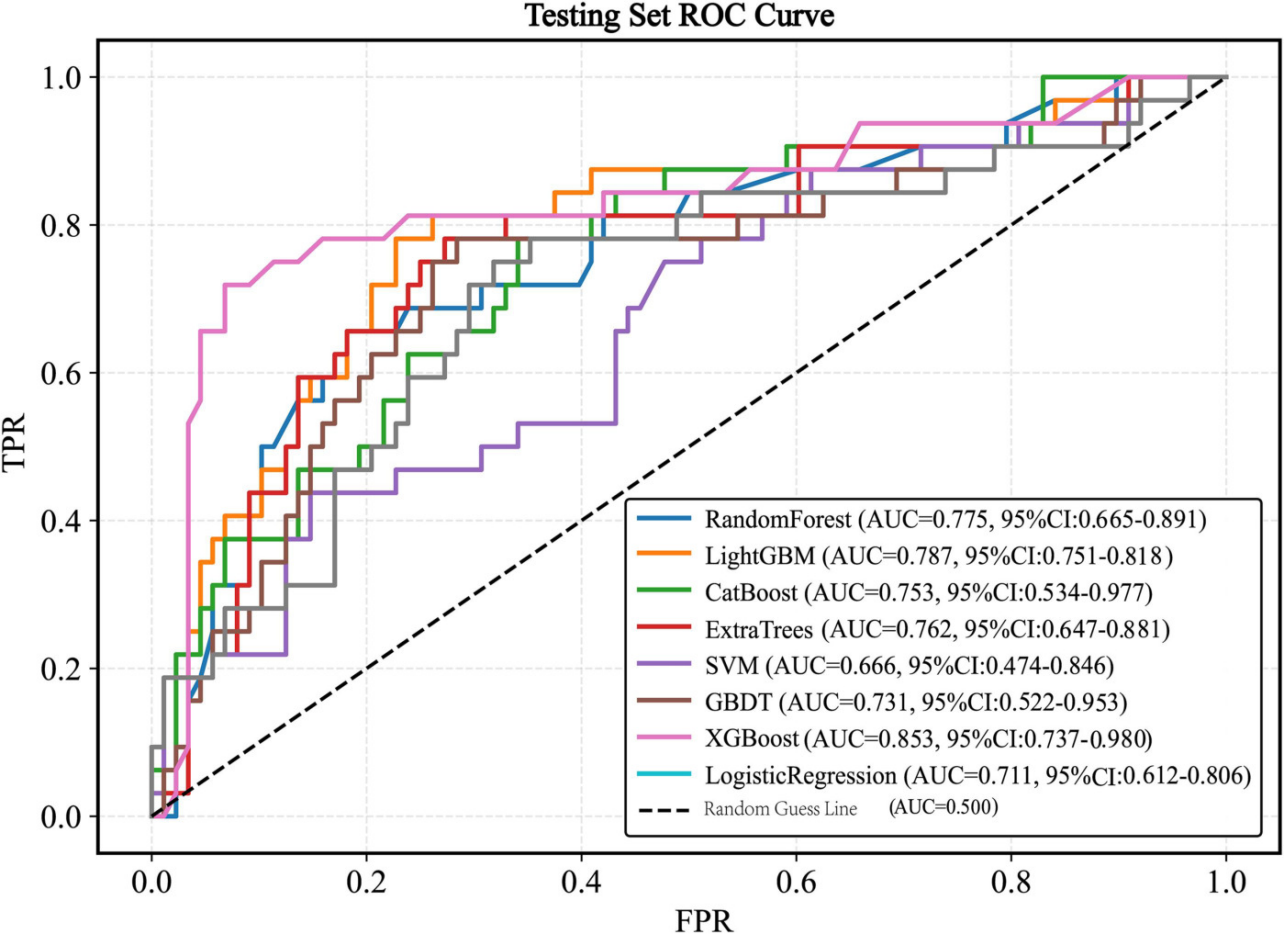
ROC curve of the test set. ROC, receiver operating characteristic; AUC, the area under the receiver operating characteristic curve; LightGBM, light gradient boosting machine; XGBoost, extreme gradient boosting; CatBoost, categorical boosting; SVM, support vector machine; GBDT, gradient boosting decision tree.

When evaluated on the testing set, the models achieved the following AUC values: XGBoost (AUC = 0.853, 95% *CI* 0.737–0.980), LightGBM (AUC = 0.787, 95% *CI* 0.751–0.818), CatBoost (AUC = 0.753, 95% *CI* 0.534–0.977), SVM (AUC = 0.666, 95% *CI* 0.474–0.846), Logistic Regression (AUC = 0.711,95%*CI* 0.612–0.806), ExtraTrees (AUC = 0.762, 95% *CI* 0.647–0.881), Random Forest (AUC = 0.775, 95% *CI* 0.665–0.891), and GBDT (AUC = 0.731, 95% *CI* 0.522–0.953). The XGBoost model exhibited the best generalization performance (Figure 6).

### 3.5 Model evaluation

**Figure 7:**
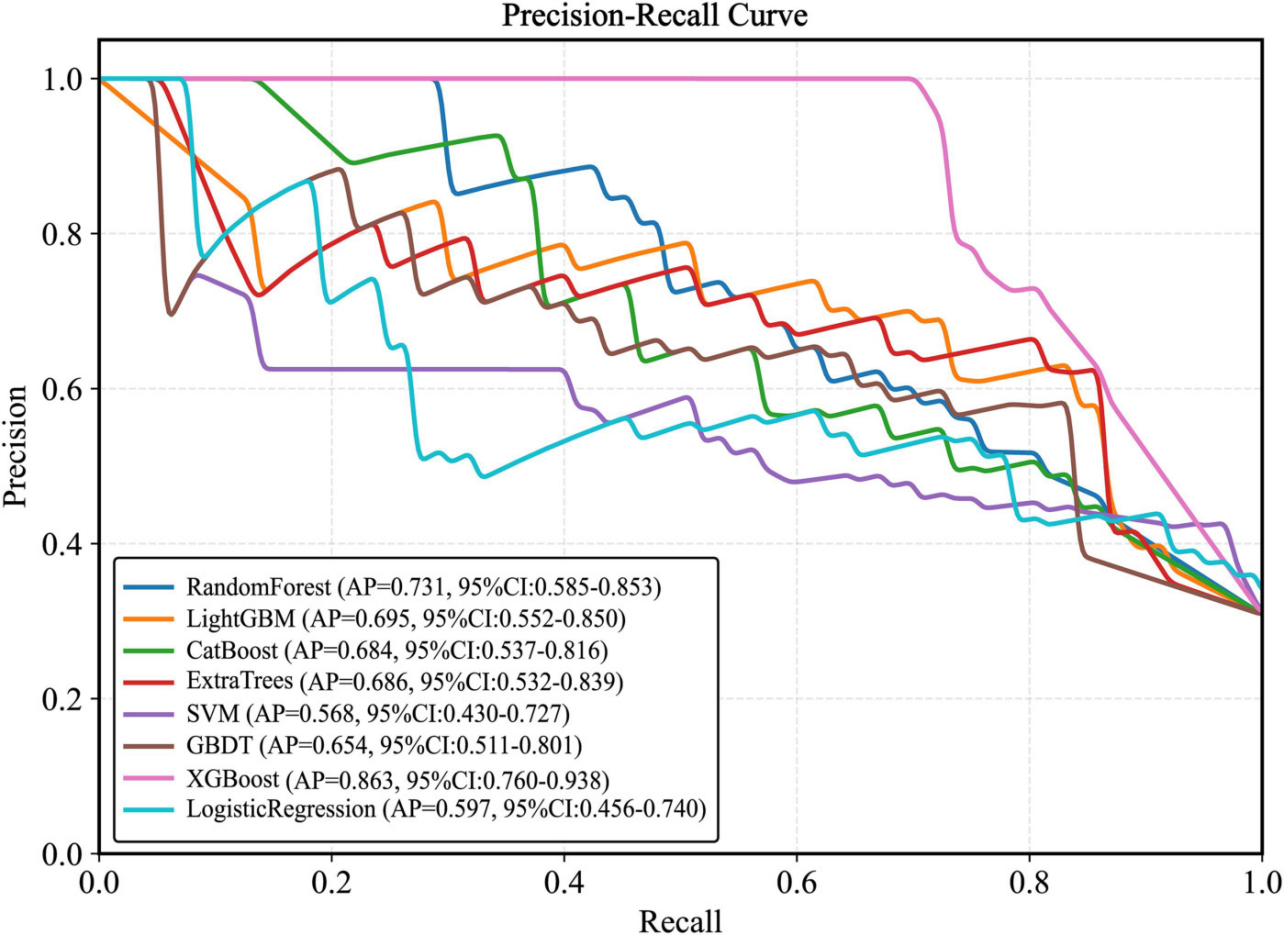
Test set precision-recall calibration smoothing curve. LightGBM, light gradient boosting machine; XGBoost, extreme gradient boosting; CatBoost, categorical boosting; SVM, support vector machine; GBDT, gradient boosting decision tree.

**Figure 8:**
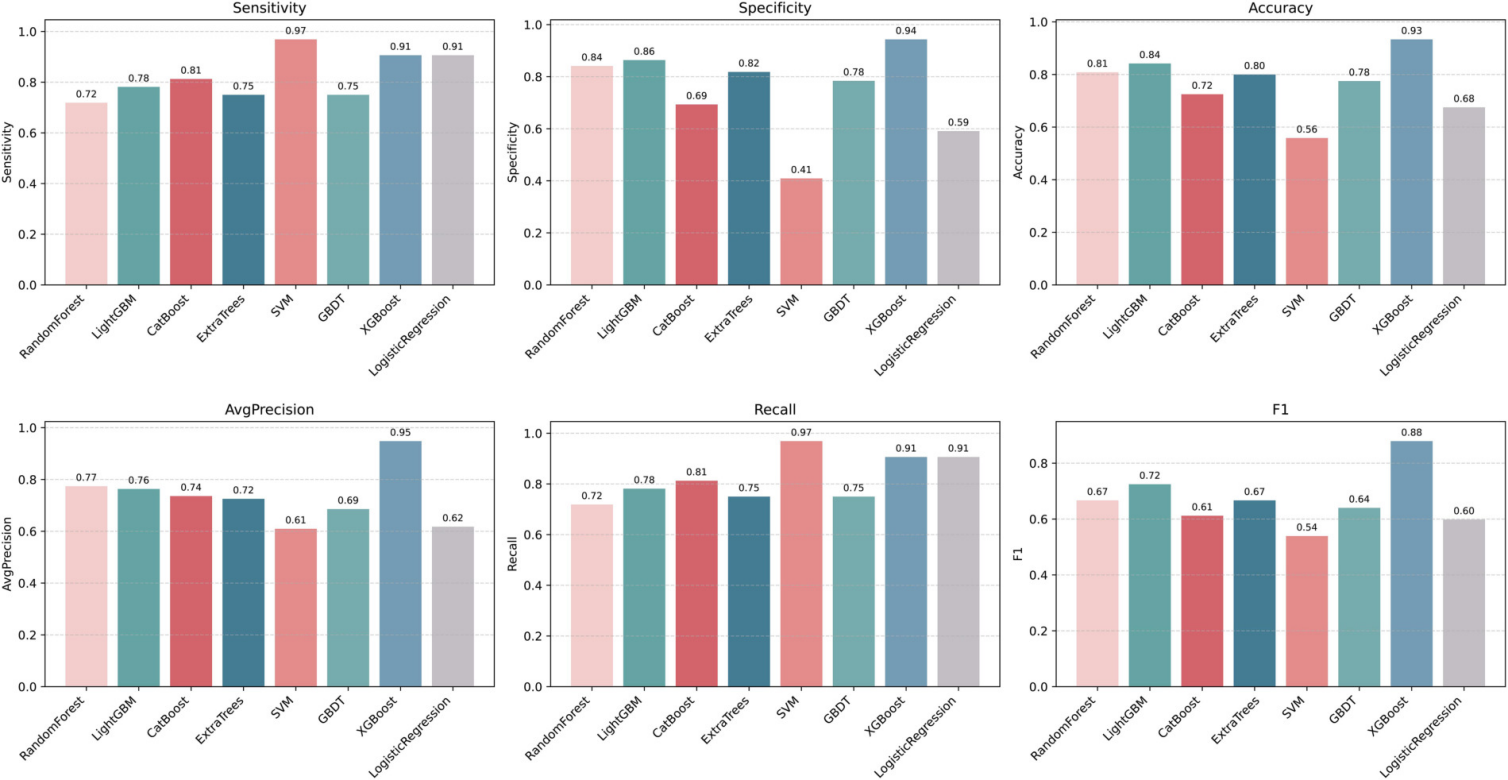
Machine learning model performance evaluation results. F1: F1score; LightGBM, light gradient boosting machine; XGBoost, extreme gradient boosting; CatBoost, categorical boosting; SVM, support vector machine; GBDT, gradient boosting decision tree.

**Figure 9:**
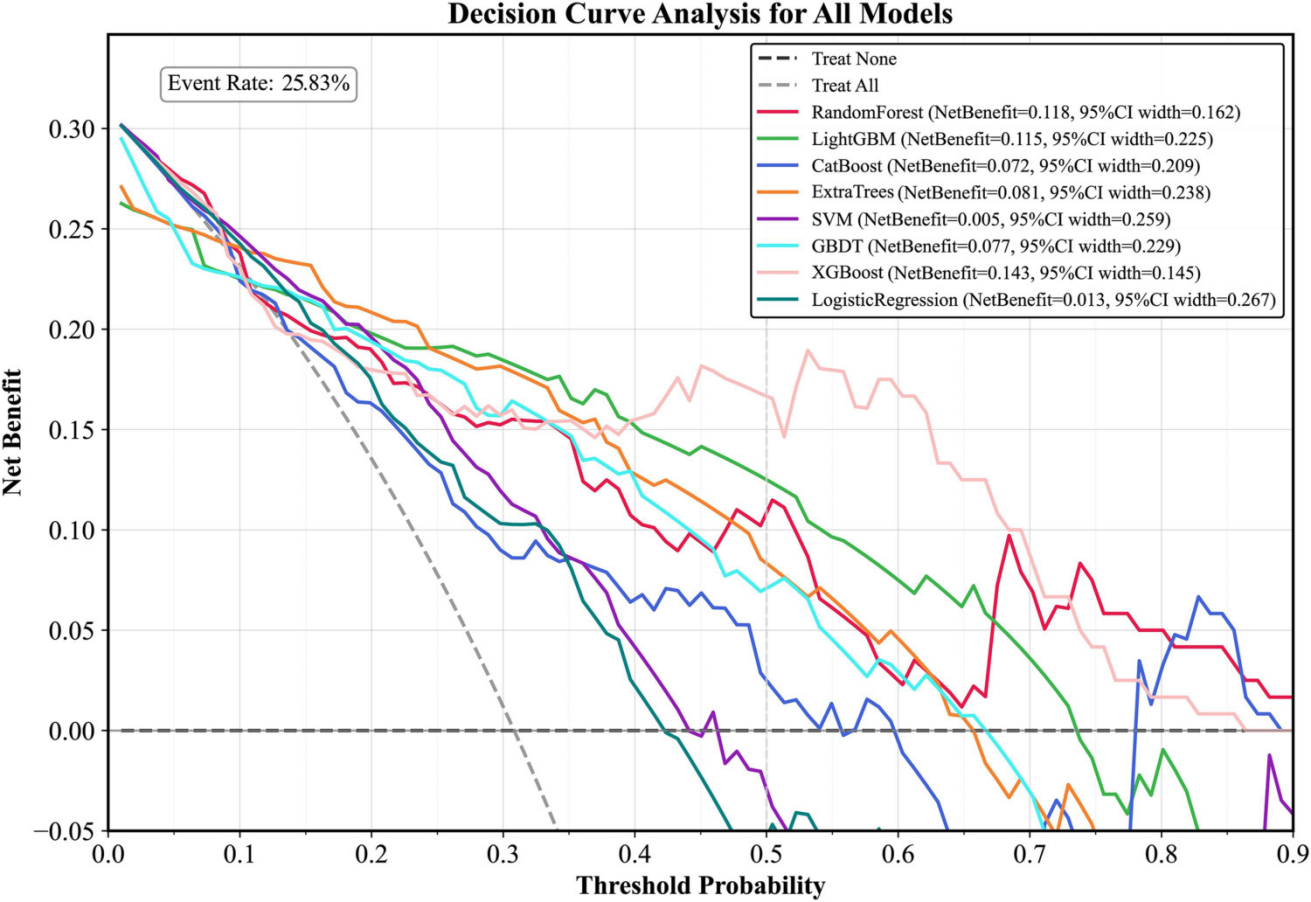
DCA curve of the test set. LightGBM, light gradient boosting machine; XGBoost, extreme gradient boosting; CatBoost, categorical boosting; SVM, support vector machine; GBDT, gradient boosting decision tree; DCA curve, decision curve analysis curve.

**Figure 10:**
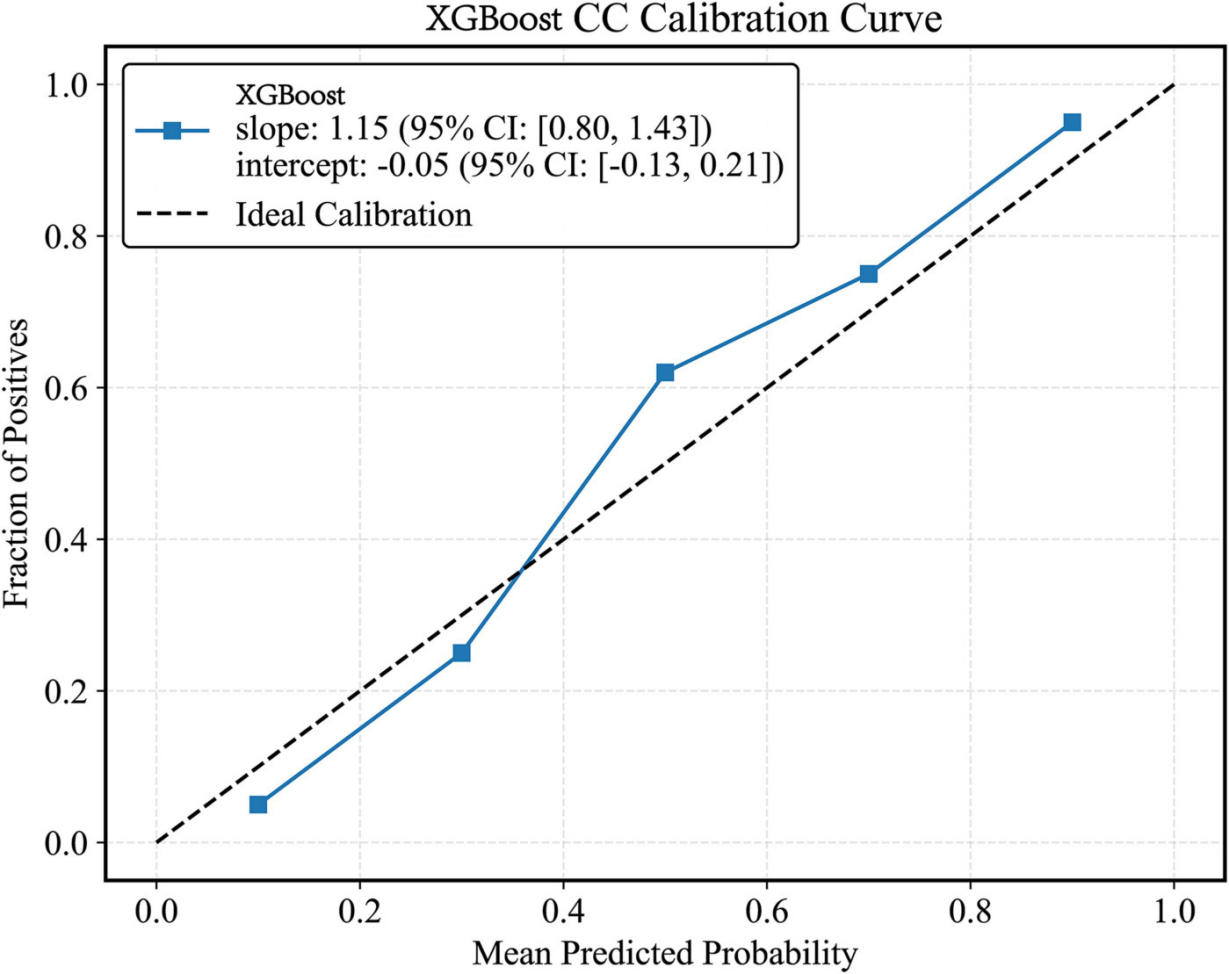
XGBoost model CC curve. CC curves, calibration curvet.

Plot the PR curve using the test set data (Figure 7). The AUC values of the PR curves for each model are as follows: XGBoost (AP = 0.875, 95% *CI* 0.760–0.938); LightGBM (AP = 0.695, 95% *CI* 0.552–0.850); CatBoost (AP = 0.684, 95% *CI* 0.537–0.816); LogisticRegression (AP = 0.597, 95% *CI* 0.456–0.740); ExtraTrees (AP = 0.686, 95% *CI* 0.532–0.839); GBDT (AP = 0.651, 95% *CI* 0.511–0.801); RandomForest (AP = 0.743, 95% *CI* 0.585–0.853); and SVM (AP = 0.568, 95% *CI* 0.430–0.727). The performance of each model was evaluated using sensitivity, specificity, accuracy, average precision, recall, and F1 score (Figure 8), and decision curve analysis (DCA) curves were plotted (Figure 9) to assess the clinical utility of the models. XGBoost demonstrated the best model performance. Using the HL goodness-of-fit test, XGBoost had *P* = 0.65, and the calibration curve is shown in Figure 10. By combining all evaluation metrics, the optimal model was determined, and the confusion matrix for the test set of the optimal model was plotted (Supplementary Figure S9) to visually demonstrate the model's predictive capability. The results showed that XGBoost was the optimal model.

### 3.6 Single-center temporal validation

For additional validation, we collected data from 82 patients between June 2025 and October 1, 2025. The ROC-AUC curve values of the XGBoost(AUC = 0.779, 95% CI 0.708–0.849) model in the validation set decreased compared to those in the test set (Supplementary Figure S10).

### 3.7 Model interpretation

**Figure 11:**
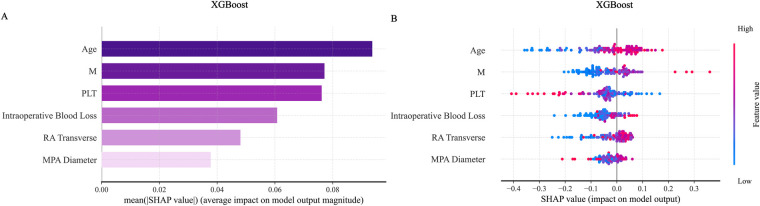
SHAP visualization graph in the XGBoost model. **(A)** Shows the SHAP importance ranking chart for the ExtraTrees model. **(B)** Shows the SHAP summary chart. SHAP, shapley additive explanations.

**Figure 12:**
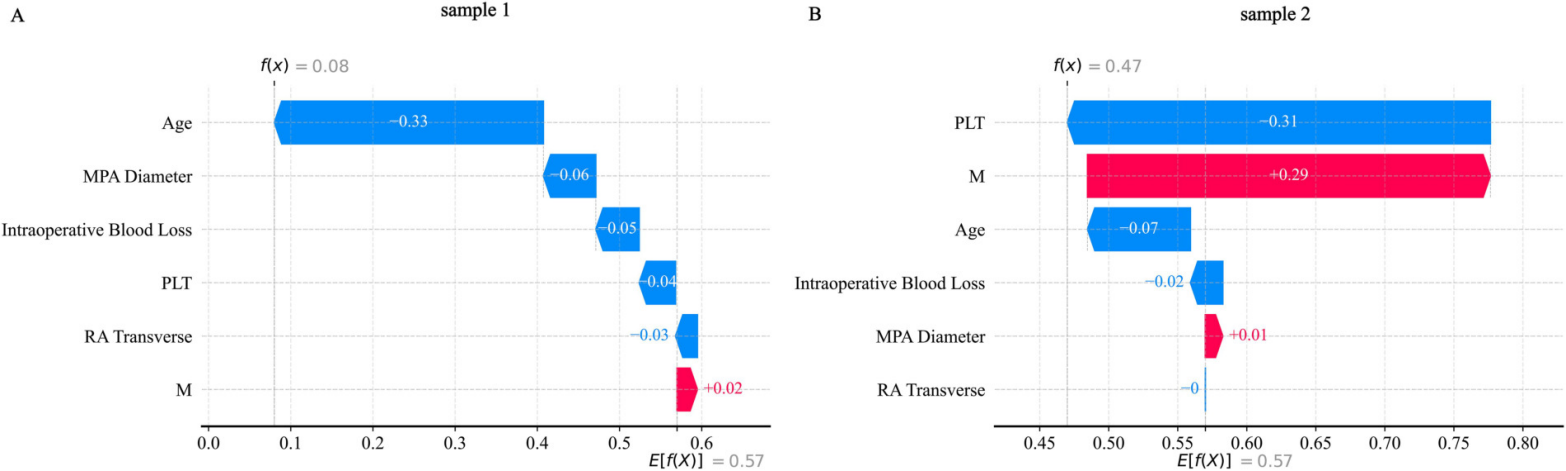
SHAP_waterfall plot. **(A)** Shows the SHAP waterfall plot for sample 1; **(B)** Shows the SHAP waterfall plot for sample 2.

SHAP analysis was applied to interpret the output of the XGBoost model. Global feature importance, assessed using SHAP bar plots (Figure 11A) and summary plots (Figure 11B), identified age and absolute monocyte count as the most influential predictors. A SHAP heatmap (Supplementary Figure S11) further illustrated feature contributions across individual samples.For detailed case analysis, two representative samples were examined: In Sample 1 (Figure 12A), with a predicted risk *f*(*x*) = 0.08, age (−0.33) and main pulmonary artery diameter (−0.06) were the primary contributors. In Sample 2 (Figure 12B; *f*(*x*) = 0.47), absolute monocyte count (+0.29) and platelet count (−0.31) had the strongest effects. Partial dependence plots (PDPs) suggested a potential synergistic interaction between age and platelet count (Supplementary Figure S1^2^).

### 3.8 Model decision threshold

Based on the precise derivation method utilizing confusion matrix counts, we established two clinically actionable decision thresholds to transform the model's predictive probabilities into practical decision tools. A high-sensitivity rule-out threshold (0.3) was defined for safely excluding disease (Sensitivity = 0.882, Specificity = 0.922, PPV = 0.652, NPV = 0.979), and a high-specificity rule-in threshold (0.4) was established for confirming high-risk status (Sensitivity = 0.765, Specificity = 0.942, PPV = 0.684, NPV = 0.960). We calculated 95% confidence intervals for the classification metrics using the Clopper-Pearson (exact) method and for the likelihood ratios using the Delta method, with complete results presented in Supplementary Table S6. The detailed clinical implementation framework and expected outcomes per 100 patients are provided in Supplementary Table S7.

## 4 Discussion

This study identified six key risk factors—Age, absolute monocyte count, right atrial transverse diameter, intraoperative blood loss, platelet count, main pulmonary artery diameter—associated with the development of ARDS within 12 h following CPB-assisted heart valve replacement surgery. These factors were selected through a hybrid approach combining the Boruta algorithm and Lasso regression. Using these predictors, eight machine learning models were developed and evaluated. Although all models demonstrated satisfactory performance, the XGBoost algorithm achieved the highest predictive accuracy (AUC = 0.853) on the test set and consistently performed well across other metrics, including sensitivity, specificity, accuracy, average precision, recall, and F1 score. Compared to traditional prediction methods in previous studies (AUC = 0.840) (22), the XGBoost model demonstrated superior predictive performance (AUC = 0.853). Among previously identified predictors, known factors such as albumin, cardiopulmonary bypass duration, and albumin levels were not classified as significant predictors in this study. Therefore, we benchmarked the XGBoost model against a minimal logistic regression model (incorporating age, albumin, time of cardiac arrest, cardiopulmonary bypass duration, and known albumin predictors, AUC = 0.803) to assess its incremental value (ΔAUC = 0.05, ΔBrier = 0.04, NRI = 0.31, IDI = 0.06). Results indicate that in our center's sample, features such as age, absolute monocyte count, right atrial transverse diameter, intraoperative blood loss, platelet count, and main pulmonary artery diameter perform better in constructing risk prediction models. This performance improvement highlights the advantages of the XGBoost gradient boosting framework: through L1/L2 regularization, loss optimization based on second-order Taylor series expansion, and efficient feature ranking, it excels at capturing complex nonlinear relationships and interactions among predictive variables. Model interpretability is further enhanced through SHAP technology, which quantifies each feature's contribution to individual prediction outcomes. Model interpretability was further enhanced using SHAP, which quantified the contribution of each feature to individual predictions.

The incidence of ARDS within 12 h postoperatively was 14% in our cohort, consistent with rates reported in previous studies (18, 23). Several recent investigations have examined risk factors for pulmonary complications after cardiac surgery. Two studies identified age, smoking history, preexisting pulmonary disease, diabetes, operative duration, CPB time, blood transfusion, and mechanical ventilation time as significant predictors of postoperative pulmonary infection (24, 25). Similarly, Jie Liu et al^.^ (26) reported that hypoalbuminemia, preoperative pulmonary dysfunction, prolonged surgery, and extended mechanical ventilation were associated with pulmonary complications. A 2024 study developed a predictive model for adverse events in elderly patients undergoing CPB cardiac surgery, though it lacked specificity for individual complications (27). Earlier efforts, such as those by Qiang Ji et al. (28), used logistic regression to identify risk factors—including age, operative time, mechanical ventilation, and transfusion—but were limited by conventional statistical approaches. Our model, by focusing on a homogeneous surgical population and a precisely defined outcome, and by incorporating novel imaging biomarkers (e.g., pulmonary artery diameter) identified through a robust feature selection process, provides a more targeted and contemporary tool for this specific clinical niche. Notably, prior studies often did not distinguish between valve and major vascular surgeries (29–31) and focused primarily on pulmonary infections or broad respiratory complications rather than early ARDS following valve replacement. This gap underscores the novelty and clinical relevance of our study.

SHAP-based interpretation of the optimal XGBoost model confirmed the importance of the selected predictors and revealed underlying interaction patterns between features.

Advanced age was reaffirmed as a significant risk factor for early ARDS, aligning with findings by Asma Zainab et al. (31). The pathogenesis of ARDS involves aberrant immune activation, wherein monocytes—key effector cells of innate immunity—play a central role. A 2017 study (32) identified elevated levels of immature CD14lowCD16^−^ monocyte subsets in patients undergoing CPB. These cells rapidly differentiate within lung tissue into TNF-α-secreting mature monocytes, precipitating acute lung injury—a mechanism corroborated by our results.

Preoperative platelet abnormalities have been linked to major adverse cardiovascular events (MACE) post-CPB (33, 34). Our findings are consistent with a 2025 study (35) highlighting the role of platelets in inflammatory cascades; altered platelet counts may reflect or contribute to postoperative inflammatory states, offering predictive insight into ARDS risk.

Significant intraoperative blood loss may promote ARDS through several pathways, including systemic hypoperfusion, impaired oxygen delivery, and activation of neuroendocrine and inflammatory responses (36). Excessive bleeding (≥100 mL) has been associated with higher rates of postoperative pulmonary complications such as pneumonia and atelectasis (36), supporting its inclusion as a key predictor in our model.

An enlarged right atrial transverse diameter often reflects right heart volume or pressure overload and may indicate right ventricular dysfunction or failure (37). Preoperative right atrial dilation can impair postoperative pulmonary perfusion and oxygenation, elevating ARDS risk (38), a observation corroborated by our analysis.

Dilation of the main pulmonary artery (≥25 mm) is a sonographic marker of pulmonary hypertension (PH), indicating chronic right ventricular afterload elevation. Patients with preoperative PH are vulnerable to ischemia-reperfusion injury during CPB, which can exacerbate pulmonary vascular resistance, right heart failure, and pulmonary edema (39). Although prior studies have not directly linked pulmonary artery dilation to ARDS, our results identify it as a salient risk factor.

The primary objective of this study was to establish and validate a robust predictive model for early postoperative acute lung injury. While SHAP analysis successfully identified and ranked clinically plausible risk factors (such as monocyte count and pulmonary artery diameter), we acknowledge that establishing precise and universally applicable intervention thresholds for these continuous variables requires further investigation. This represents a common challenge in the early stages of clinical prediction model development, as threshold determination depends not only on model outputs but also requires integration with clinical cost-benefit analysis and external validation in larger, more diverse cohorts.

The dual-threshold decision framework proposed in this study can serve as a reference for clinical action.The low threshold (0.3) is used for “exclusion”. In large-scale screenings with limited resources, individuals with a predicted probability below 0.3 can be considered low-risk, effectively reducing unnecessary follow-up examinations and optimizing resource allocation. The high threshold (0.4) is used for “inclusion”: Individuals with a predicted probability above 0.4 demonstrate a significantly increased likelihood of having the target disease. This outcome provides strong decision support for clinicians, recommending more proactive and thorough diagnostic evaluation or early intervention for such patients, thereby enabling early diagnosis and treatment.

The demonstrated superiority of XGBoost over traditional logistic regression underscores the value of machine learning approaches in capturing complex clinical relationships. Based on our findings, we recommend that clinicians vigilantly monitor elderly patients presenting with preoperative right atrial enlargement, pulmonary artery dilation, elevated monocyte or platelet counts, or anticipated significant intraoperative blood loss. Early identification of high-risk individuals may facilitate proactive ICU management and preventive strategies, ultimately improving postoperative outcomes.

While this model was developed and demonstrates promising performance within our institutional cohort, its generalizability to other medical centers requires careful consideration. The model's transportability may be influenced by variations in patient demographics, surgical protocols, and perioperative care standards across different healthcare settings. Although we employed a temporal validation approach within our single-center dataset to enhance methodological rigor—demonstrating preserved discrimination (AUC 0.779) and acceptable calibration following simple recalibration—this represents an intermediate step in the validation hierarchy. The observed performance attenuation in temporal validation, while expected and correctable through recalibration, underscores the necessity of external validation in truly independent populations. Future work should therefore prioritize prospective multicenter collaboration to rigorously evaluate model performance across diverse clinical environments. Such external validation constitutes an essential prerequisite for meaningful clinical implementation and will inform any necessary model refinement for broader application.

## 5 Limitations

This study has several limitations that should be considered when interpreting the results: (1) The sample size was relatively limited, which may affect the stability of feature selection and increase the risk of model overfitting. (2) The retrospective collection of medical history and clinical variables may be subject to information bias, including recall bias and incomplete documentation, potentially influencing the reliability of the data. (3) The inclusion of unclassified or heterogeneous types of valvular heart disease may introduce unmeasured confounding factors. (4) Although validated through a single-center time series, the lack of multi-center external validation cohorts limits the model's generalizability. Future studies should validate and optimize this model through larger-scale multi-center cohort research. (5) Furthermore, this study is constrained by retrospective data, and certain inflammatory markers potentially associated with ARDS (e.g., CRP, IL-6) were excluded from analysis due to data gaps resulting from their non-routine preoperative testing. Future prospective studies should incorporate these indicators to further validate and enrich the model's biological foundation.

## 6 Conclusions

This study developed and evaluated multiple machine learning models using clinical data from patients who underwent CPB-assisted cardiac valve replacement surgery at the Department of Cardiovascular Surgery, First Hospital of Lanzhou University, between January 2023 and February 2025, to predict ARDS following the procedure. The XGBoost model demonstrated superior performance, indicating its considerable potential for clinical application. Several key factors—Age, absolute monocyte count,right atrial transverse diameter, intraoperative blood loss, platelet count, main pulmonary artery diameter—were identified as key predictors of postoperative ARDS. These findings hold significant clinical relevance for early identification of high-risk patients and improved perioperative management in individuals undergoing CPB-assisted heart valve replacement.

## Data Availability

The original contributions presented in the study are included in the article/Supplementary Material, further inquiries can be directed to the corresponding authors.
