# Supplementary material for: Machine learning prediction of ARDS after heart valve surgery: development and validation in Northwest China
Source: Front Cardiovasc Med. 2026 Jan 21;12:1696326. doi: 10.3389/fcvm.2025.1696326 (PMC12868288; doi:10.3389/fcvm.2025.1696326)
Supplement: Supplementary file 1 [file Table1.docx]

| Time Period | Incidence Rate(%) | AUC | Accuracy | | | AvgPrecision | Recall |
| --- | --- | --- | --- | --- | --- | --- | --- |
| 12h | 14.00 | 0.853 | | | 0.930 | 0.950 | 0.910 |
| 24h | 12.80 | 0.842 | | 0.930 | | 0.920 | 0.820 |
| 48h | 11.95 | 0.785 | | 0.890 | | 0.860 | 0.780 |
