# Supplementary material for: Machine learning prediction of ARDS after heart valve surgery: development and validation in Northwest China
Source: Front Cardiovasc Med. 2026 Jan 21;12:1696326. doi: 10.3389/fcvm.2025.1696326 (PMC12868288; doi:10.3389/fcvm.2025.1696326)
Supplement: Supplementary file 2 [file Table2.docx]

| Variables | | Missing Ratio(%) | |
| --- | --- | --- | --- |
| Sex | | 0.50 | |
| Age | | 0.50 | |
| BMI | | 0.50 | |
| Smoke | | 0.50 | |
| Alcohol | | 0.50 | |
| Hypertension | | 0.50 | |
| CHD | | 9.70 | |
| MI | | 0.70 | |
| AF | | 0.70 | |
| Diabetes | | 0.70 | |
| CRP | | 36.60 | |
| HB | | 1.00 | |
| WBC | | 1.00 | |
| PLT | | 1.00 | |
| RBC | | 1.00 | |
| N | | 1.00 | |
| L | | 1.00 | |
| M | | 1.00 | |
| Eosinophils | | 1.00 | |
| Basophils | | 1.20 | |
| AST | | 2.00 | |
| ALT | | 2.00 | |
| Total Bilirubin | | 2.00 | |
| Direct Bilirubin | | 2.00 | |
| Indirect Bilirubin | | 2.00 | |
| Total Protein | | 2.70 | |
| Globulin | | 2.50 | |
| Albumin | | 2.20 | |
| Serum Creatinine | | 2.20 | |
| Urea | | 2.20 | |
| K | 31.80 | |  |
| Na | 31.80 | |  |
| CL | 31.80 | |  |
| Ca | 31.80 | |  |
| Fasting Blood Glucose | 31.80 | |  |
| Total Cholesterol | 2.70 | |  |
| Triglycerides | 2.70 | |  |
| HDL | 2.70 | |  |
| LDL | 2.70 | |  |
| LDH | 2.70 | |  |
| Homocysteine | 3.20 | |  |
| CK | 3.00 | |  |
| BNP | 33.60 | |  |
| INR | 2.00 | |  |
| PT | 2.00 | |  |
| APTT | 3.00 | |  |
| D-Dimer | 29.90 | |  |
| FDP | 30.10 | |  |
| FIB | 2.50 | |  |
| TT | 7.00 | |  |
| FiO₂ | 33.10 | |  |
| PO₂ | 32.10 | |  |
| PaCO₂ | 31.60 | |  |
| SaO₂ | 32.10 | |  |
| PH | 31.80 | |  |
| BE | 32.80 | |  |
| K⁺ | 31.80 | |  |
| Na⁺ | 31.80 | |  |
| Ca²⁺ | 31.80 | |  |
| Lac | 31.80 | |  |
| Hct | 32.10 | |  |
| THbc | 32.20 | |  |
| AV Annulus | 1.00 | |  |
| Aortic Sinus | 42.30 | |  |
| Ascending Aorta | 45.00 | |  |
| LAAP | 0.70 | |  |
| RVAP | 0.70 | |  |
| RVOT | 0.70 | |  |
| MPA Diameter | 0.70 | |  |
| LVEDD | 0.50 | |  |
| LVESD | 0.50 | |  |
| LVED-LR | 0.50 | |  |
| LVES-LR | 0.50 | |  |
| LVED | 0.50 | |  |
| LVES | 0.50 | |  |
| RV Long | 0.50 | |  |
| RV Transverse | 0.50 | |  |
| LA Long | 0.50 | |  |
| LA Transverse | 0.50 | |  |
| RA Long | 0.50 | |  |
| RA Transverse | 0.50 | |  |
| LVEDV | 42.00 | |  |
| LVEF | 0.50 | |  |
| FS | 4.00 | |  |
| Mean AV Gradient | 38.10 | |  |
| AV Peak Velocity | 37.30 | |  |
| AR Grade | 43.00 | |  |
| Mean MV Gradient | 44.50 | |  |
| MV Peak Velocity | 55.50 | |  |
| MVA | 62.20 | |  |
| MV Annulus | 39.30 | |  |
| MR Grade | 49.50 | |  |
| Mean TV Gradient | 40.30 | |  |
| TV Peak Velocity | 43.50 | |  |
| TVA | 72.90 | |  |
| TR Grade | 59.50 | |  |
| PASP | 60.70 | |  |
| Mean PV Gradient | 55.50 | |  |
| PV Peak Velocity | 68.20 | |  |
| PV Regurgitation Grade | 58.70 | |  |
| DR Inflammatory Changes | 0.50 | |  |
| DR Patchy Exudative | 0.50 | |  |
| CT Inflammatory | 41.00 | |  |
| CT Patchy Exudative | 40.30 | |  |
| Total Time | 1.70 | |  |
| Full Bypass Time | 1.50 | |  |
| Partial Bypass Time | 1.50 | |  |
| Cardiac Arrest Time | 1.50 | |  |
| Rewarming Time | 1.50 | |  |
| Intraoperative Blood Loss | 4.00 | |  |
| Plasma Transfusion | 0.00 | |  |
| RBC Transfusion | 0.00 | |  |
| PLT Transfusion | 0.00 | |  |
| Cryoprecipitate | 0.00 | |  |
| Intraoperative Ultrafiltration | 14.40 | |  |
| Urine Output | | 31.30 | |
