# Supplementary material for: Machine learning prediction of ARDS after heart valve surgery: development and validation in Northwest China
Source: Front Cardiovasc Med. 2026 Jan 21;12:1696326. doi: 10.3389/fcvm.2025.1696326 (PMC12868288; doi:10.3389/fcvm.2025.1696326)
Supplement: Supplementary file 3 [file Table3.docx]

| Subgroup | AUC | Sensitivity | | Specificity | Accuracy | AvgPrecision |
| --- | --- | --- | --- | --- | --- | --- |
| Sex | | | | | | |
| Male（n=66） | 0.842 | 0.952 | 0.900 | | 0.967 | 0.969 |
| Female（n=54） | 0.846 | 0.952 | 0.963 | | 0.958 | 0.954 |
| Age(year) | | | | | | |
| ≤65（n=105） | 0.826 | 0.909 | 0.870 | | 0.962 | 0.960 |
| Total time（min） | | | | | | |
| Total_Time_Low(n=40) | 0.801 | 0.918 | 0.833 | | 0.86 | 0.867 |
| Total_Time_Medium(n=40) | 0.812 | 0.847 | 0.876 | | 0.866 | 0.773 |
| Total_Time_High(n=40) | 0.743 | 0.869 | 0.745 | | 0.600 | 0.747 |
| Location of valvular lesions | | | | | | |
| Aortic valve(62) | 0.716 | 0.907 | 0.765 | | 0.714 | 0.75 |
| Mitral valve(47) | 0.703 | 0.858 | 0.702 | | 0.765 | 0.622 |
