# Supplementary material for: Machine learning prediction of ARDS after heart valve surgery: development and validation in Northwest China
Source: Front Cardiovasc Med. 2026 Jan 21;12:1696326. doi: 10.3389/fcvm.2025.1696326 (PMC12868288; doi:10.3389/fcvm.2025.1696326)
Supplement: Supplementary file 4 [file Table4.docx]

|  | Testing Set AUC(95% CI) |
| --- | --- |
| (i) pre-op only | 0.798 (0.732-0.864) |
| (ii) pre-op + intra-op | 0.853 (0.795-0.911) |
| (iii) early ICU (first hour) | - |
