# Supplementary material for: Machine learning prediction of ARDS after heart valve surgery: development and validation in Northwest China
Source: Front Cardiovasc Med. 2026 Jan 21;12:1696326. doi: 10.3389/fcvm.2025.1696326 (PMC12868288; doi:10.3389/fcvm.2025.1696326)
Supplement: Supplementary file 5 [file Table5.docx]

| Variable | Original Mean | Original Std | Imputed Mean | | | Imputed Std | | SMD |
| --- | --- | --- | --- | --- | --- | --- | --- | --- |
| Age | 53.10 | 10.77 | | 53.09 | 10.75 | | ＜0.1 | |
| BMI | 23.10 | 3.17 | | 23.10 | 3.16 | | ＜0.1 | |
| HB | 145.15 | 54.43 | | 145.22 | 54.17 | | ＜0.1 | |
| WBC | 5.66 | 2.19 | | 5.66 | 2.18 | | ＜0.1 | |
| PLT | 184.55 | 60.17 | | 184.55 | 59.86 | | ＜0.1 | |
| RBC | 4.69 | 0.58 | | 4.69 | 0.58 | | ＜0.1 | |
| N | 4.60 | 9.16 | | 4.59 | 9.12 | | ＜0.1 | |
| L | 2.26 | 4.58 | | 2.26 | 4.55 | | ＜0.1 | |
| M | 0.53 | 1.10 | | 0.53 | 1.10 | | ＜0.1 | |
| Eosinophils | 0.15 | 0.31 | | 0.15 | 0.31 | | ＜0.1 | |
| Basophils | 0.04 | 0.06 | | 0.04 | 0.06 | | ＜0.1 | |
| AST | 28.96 | 34.06 | | 29.06 | 33.75 | | ＜0.1 | |
| ALT | 30.01 | 46.11 | | 30.12 | 45.67 | | ＜0.1 | |
| Total Bilirubin | 18.32 | 9.21 | | 18.31 | 9.12 | | ＜0.1 | |
| Direct Bilirubin | 3.73 | 2.26 | | 3.74 | 2.24 | | ＜0.1 | |
| Indirect Bilirubin | 14.56 | 7.37 | | 14.54 | 7.30 | | ＜0.1 | |
| Total Protein | 72.64 | 57.07 | | 72.63 | 56.36 | | ＜0.1 | |
| Globulin | 27.12 | 7.15 | | 27.12 | 7.06 | | ＜0.1 | |
| Albumin | 43.14 | 17.96 | | 43.13 | 17.76 | | ＜0.1 | |
| Serum Creatinine | 74.71 | 28.99 | | 74.73 | 28.67 | | ＜0.1 | |
| Urea | 6.85 | 4.09 | | 6.85 | 4.05 | | ＜0.1 | |
| Total Cholesterol | 3.98 | 1.01 | | 3.96 | 1.02 | | ＜0.1 | |
| Triglycerides | 1.42 | 0.92 | | 1.42 | 0.92 | | ＜0.1 | |
| HDL | 1.07 | 0.27 | | 1.07 | 0.28 | | ＜0.1 | |
| LDL | 2.55 | 0.75 | | 2.54 | 0.75 | | ＜0.1 | |
| LDH | 224.99 | 200.93 | | 225.01 | 198.15 | | ＜0.1 | |
| Homocysteine | 18.15 | 13.64 | | 18.13 | 13.43 | | ＜0.1 | |
| CK | 77.06 | 101.22 | | 77.38 | 99.88 | | ＜0.1 | |
| INR | 1.20 | 0.55 | | 1.20 | 0.54 | | ＜0.1 | |
| PT | 13.17 | 6.15 | | 13.14 | 6.11 | | ＜0.1 | |
| APTT | 34.33 | 16.03 | | 34.33 | 15.79 | | ＜0.1 | |
| D-Dimer | 0.65 | 2.02 | | 0.63 | 1.75 | | ＜0.1 | |
| FIB | 2.87 | 0.73 | | 2.87 | 0.72 | | ＜0.1 | |
| TT | 13.98 | 2.88 | | 13.97 | 2.79 | | ＜0.1 | |
| AV Annulus | 23.15 | 3.52 | | 23.18 | 3.58 | | ＜0.1 | |
| LAAP | 42.92 | 18.56 | | 42.92 | 18.49 | | ＜0.1 | |
| RVAP | 27.30 | 4.58 | | 27.30 | 4.56 | | ＜0.1 | |
| RVOT | 29.47 | 3.88 | | 29.51 | 3.93 | | ＜0.1 | |
| MPA Diameter | 28.21 | 14.76 | | 28.21 | 14.70 | | ＜0.1 | |
| LVEDD | 55.81 | 8.28 | | 55.81 | 8.26 | | ＜0.1 | |
| LVESD | 40.20 | 7.75 | | 40.20 | 7.74 | | ＜0.1 | |
| LVED-LR | 55.89 | 8.25 | | 55.88 | 8.23 | | ＜0.1 | |
| LVES-LR | 40.23 | 7.75 | | 40.23 | 7.73 | | ＜0.1 | |
| LVED | 81.82 | 10.83 | | 81.83 | 10.80 | | ＜0.1 | |
| LVES | 67.29 | 10.38 | | 67.30 | 10.35 | | ＜0.1 | |
| RV Long | 66.49 | 8.57 | | 66.49 | 8.55 | | ＜0.1 | |
| RV Transverse | 32.50 | 5.38 | | 32.50 | 5.37 | | ＜0.1 | |
| LA Long | 63.06 | 15.57 | | 63.06 | 15.53 | | ＜0.1 | |
| LA Transverse | 50.91 | 12.66 | | 50.90 | 12.63 | | ＜0.1 | |
| RA Long | 55.45 | 51.34 | | 55.43 | 51.21 | | ＜0.1 | |
| RA Transverse | 43.15 | 25.70 | | 43.14 | 25.63 | | ＜0.1 | |
| LVEF | 57.59 | 25.64 | | 57.59 | 25.57 | | ＜0.1 | |
| FS | 29.00 | 4.07 | | 28.94 | 4.07 | | ＜0.1 | |
| total time | 141.20 | 62.14 | | 141.00 | 61.75 | | ＜0.1 | |
| Full Bypass Time | 100.80 | 48.58 | | 100.61 | 48.33 | | ＜0.1 | |
| Partial Bypass Time | 39.57 | 23.75 | | 39.47 | 23.61 | | ＜0.1 | |
| Cardiac Arrest Time | 104.70 | 52.01 | | 104.54 | 51.71 | | ＜0.1 | |
| Rewarming Time | 86.40 | 46.76 | | 86.24 | 46.49 | | ＜0.1 | |
| Intraoperative Blood Loss | 353.52 | 215.77 | | 352.60 | 211.67 | | ＜0.1 | |
| Intraoperative Ultrafiltration | 1650.87 | 950.76 | | 1640.94 | 888.45 | | ＜0.1 | |
| Sex | 0.45 | 0.50 | | 0.45 | 0.50 | | ＜0.1 | |
| Smoke | 0.10 | 0.29 | | 0.09 | 0.29 | | ＜0.1 | |
| Alcohol | 0.08 | 0.27 | | 0.08 | 0.27 | | ＜0.1 | |
| Hypertension | 0.22 | 0.41 | | 0.22 | 0.41 | | ＜0.1 | |
| CHD | 0.35 | 0.48 | | 0.32 | 0.47 | | ＜0.1 | |
| MI | 0.02 | 0.14 | | 0.02 | 0.14 | | ＜0.1 | |
| AF | 0.25 | 0.43 | | 0.24 | 0.43 | | ＜0.1 | |
| Diabetes | 0.06 | 0.23 | | 0.05 | 0.23 | | ＜0.1 | |
| DR Inflammatory Changes | 0.09 | 0.28 | | 0.09 | 0.28 | | ＜0.1 | |
| DR Patchy Exudative | 0.10 | 0.29 | | 0.09 | 0.29 | | ＜0.1 | |
