# Supplementary material for: Machine learning prediction of ARDS after heart valve surgery: development and validation in Northwest China
Source: Front Cardiovasc Med. 2026 Jan 21;12:1696326. doi: 10.3389/fcvm.2025.1696326 (PMC12868288; doi:10.3389/fcvm.2025.1696326)
Supplement: Supplementary file 6 [file Table6.docx]

| Threshold | Metric | Value (95% CI) | | LR+ (95% CI) | LR- (95% CI) |
| --- | --- | --- | --- | --- | --- |
| 0.3 | Sensitivity | 0.882 (0.676, 0.971) | 7.94 (4.02, 15.67) | | 0.13 (0.04, 0.46) |
|  | Specificity | 0.922 (0.856, 0.963) |  | |  |
|  | PPV | 0.652 (0.451, 0.816) |  | |  |
|  | NPV | 0.979 (0.929, 0.996) |  | |  |
| 0.4 | Sensitivity | 0.765 (0.543, 0.911) | 8.83 (4.12, 18.92) | | 0.25 (0.11, 0.56) |
|  | Specificity | 0.942 (0.882, 0.975) |  | |  |
|  | PPV | 0.684 (0.469, 0.849) |  | |  |
|  | NPV | 0.960 (0.904, 0.986) |  | |  |
