# Supplementary material for: Machine learning prediction of ARDS after heart valve surgery: development and validation in Northwest China
Source: Front Cardiovasc Med. 2026 Jan 21;12:1696326. doi: 10.3389/fcvm.2025.1696326 (PMC12868288; doi:10.3389/fcvm.2025.1696326)
Supplement: Supplementary file 7 [file Table7.docx]

| Risk Stratification | Threshold | Patients | Key Performance Metrics (95% CI) | Clinical Interpretation & Action Recommendations |
| --- | --- | --- | --- | --- |
| Low Risk (Rule-Out) | < 0.30 | ~85 patients | Sensitivity: 0.882 (0.676, 0.971);NPV: 0.979 (0.929, 0.996);LR-: 0.13 (0.04, 0.46) | Clinical Interpretation: This threshold has a very low miss rate (~1.2%) and a high negative predictive value. It can be used to rule out ARDS in low-risk patients.  Action Recommendations: Admit to a standard monitoring ward under routine postoperative management;Reduce unnecessary frequent blood gas analyses and chest imaging to optimize resource utilization |
| Intermediate Risk (Observe) | 0.30 – 0.39 | ~8 patients | "gray zone" | Clinical Interpretation: Patients are at a borderline risk level and require careful assessment.  Action Recommendations:Recommend enhanced monitoring (e.g., increased frequency of respiratory rate and SpO₂ checks) in an ICU or step-down unit;Be vigilant for any potential triggering factors (e.g., fluid overload, signs of infection). |
|  |  |  |  |  |
| High Risk (Rule-In) | ≥ 0.40 | ~15 patients | Specificity: 0.942 (0.882, 0.975);PPV: 0.684 (0.469, 0.849);LR+: 8.83 (4.12, 18.92) | Clinical Interpretation: This threshold has a low false alarm rate and a moderate positive predictive value. It can be used to identify truly high-risk patients.  Action Recommendations:Trigger preventive lung-protective strategies: Consider low tidal volume ventilation, aggressive diuresis, conservative transfusion strategy, and avoidance of fluid overload;Enhance airway management and prepare respiratory support equipment preemptively.  unnecessary frequent blood gas analyses and chest imaging to optimize resource utilization |
