# Supplementary figures and images for: Machine learning prediction of ARDS after heart valve surgery: development and validation in Northwest China
Source: Front Cardiovasc Med. 2026 Jan 21;12:1696326. doi: 10.3389/fcvm.2025.1696326 (PMC12868288; doi:10.3389/fcvm.2025.1696326)

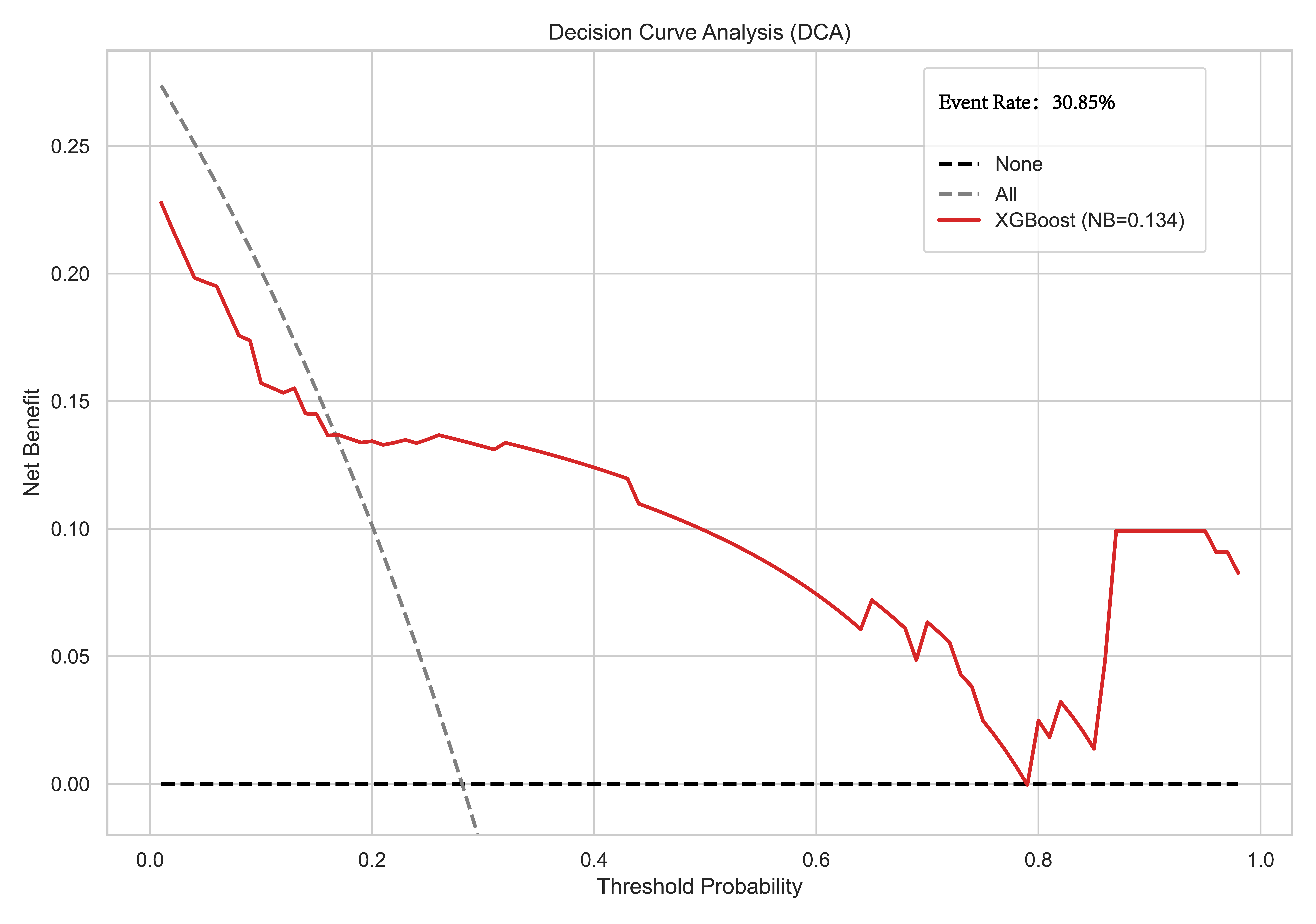

Supplement: Supplementary file 8 [file Image15.tif]

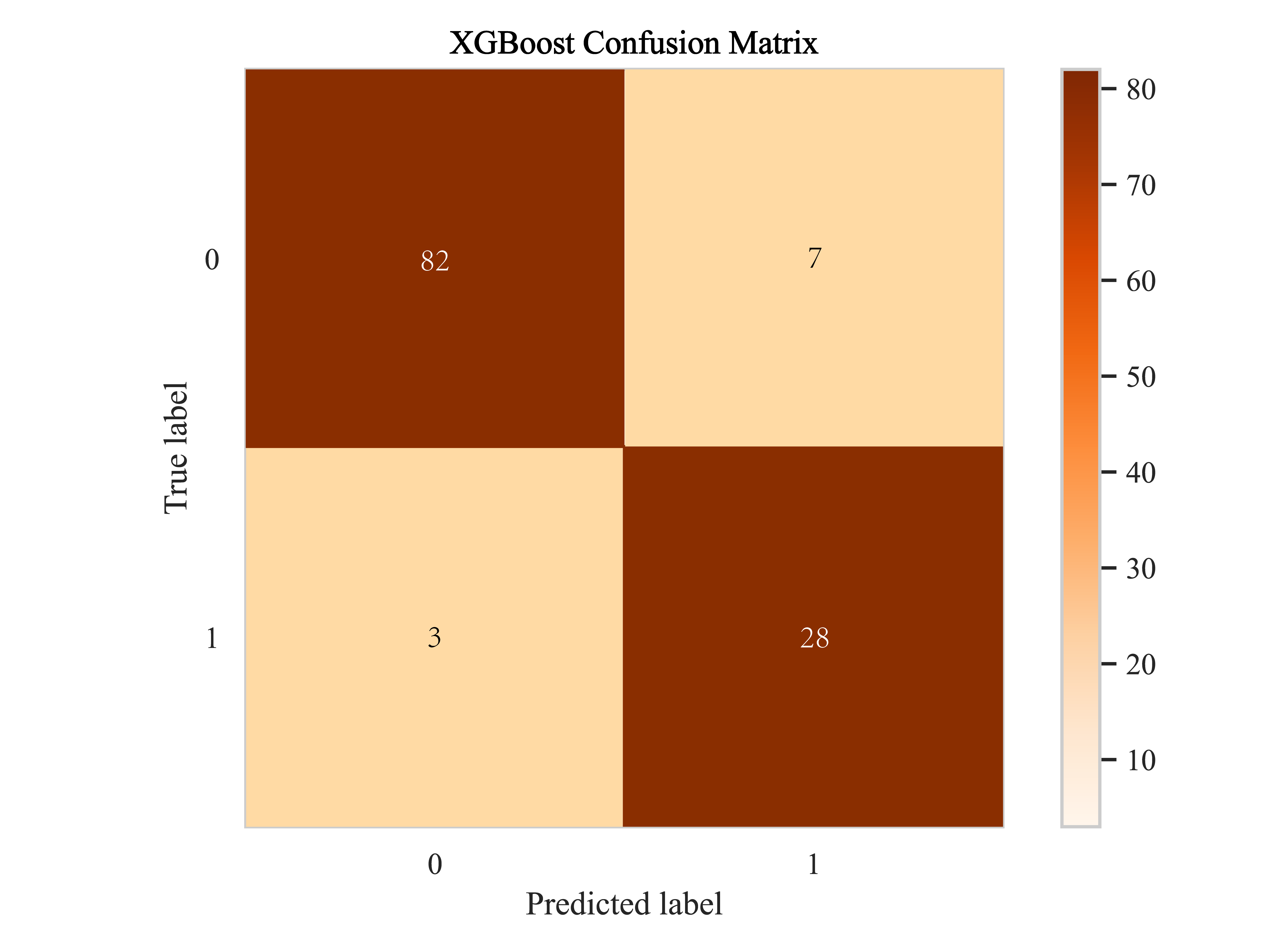

Supplement: Supplementary file 17 [file Image9.png]

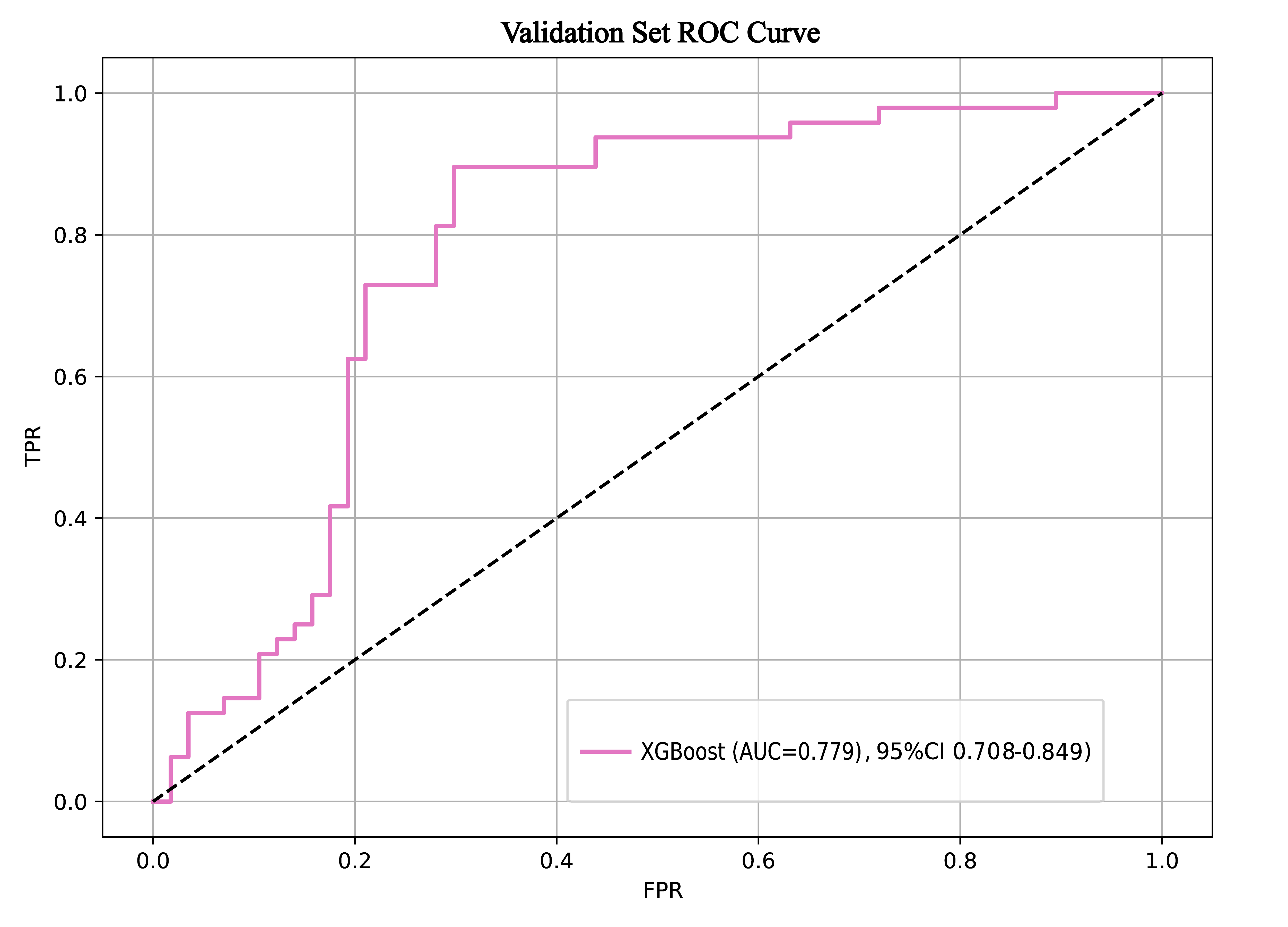

Supplement: Supplementary file 18 [file Image10.png]

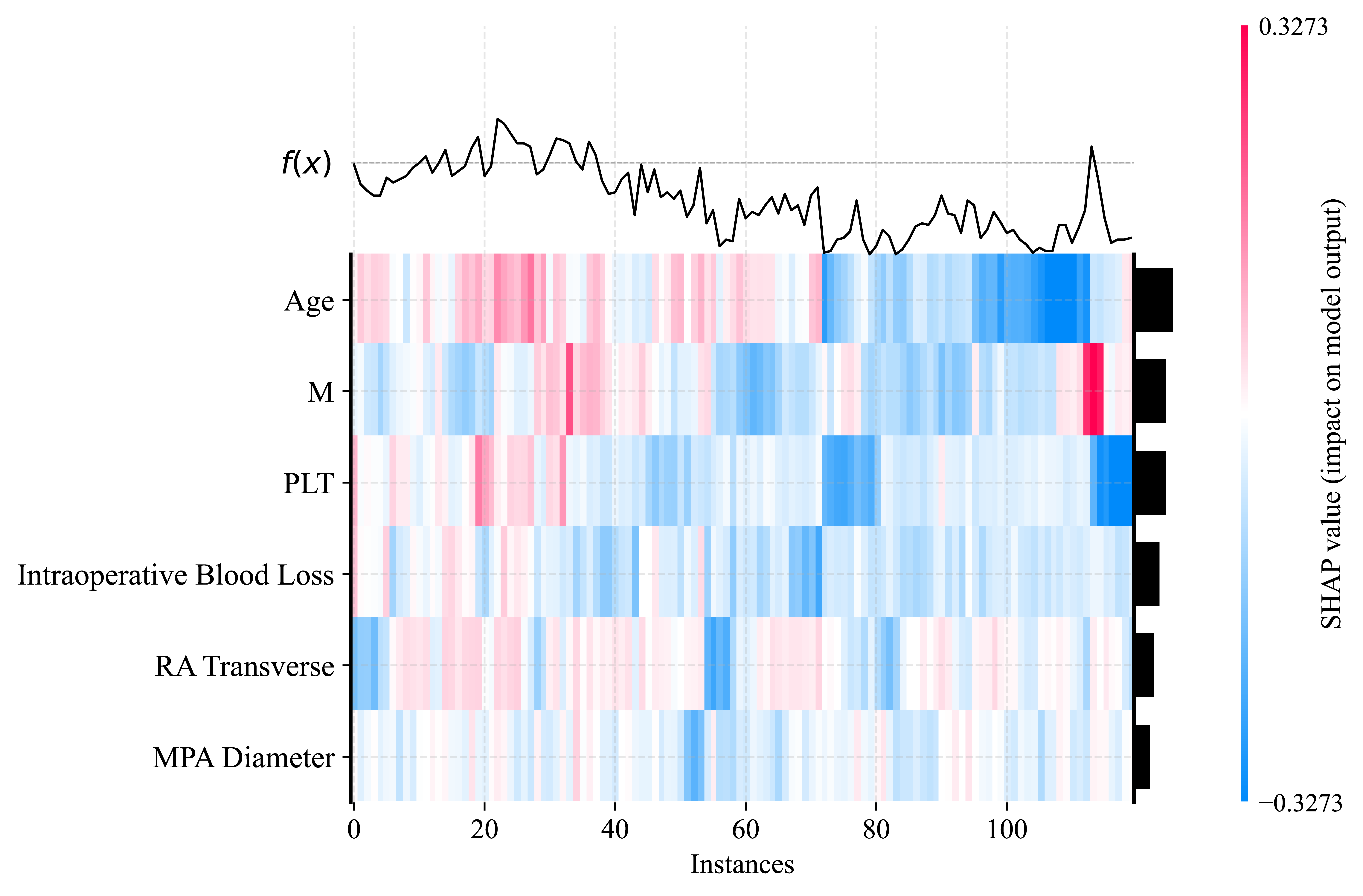

Supplement: Supplementary file 19 [file Image11.png]

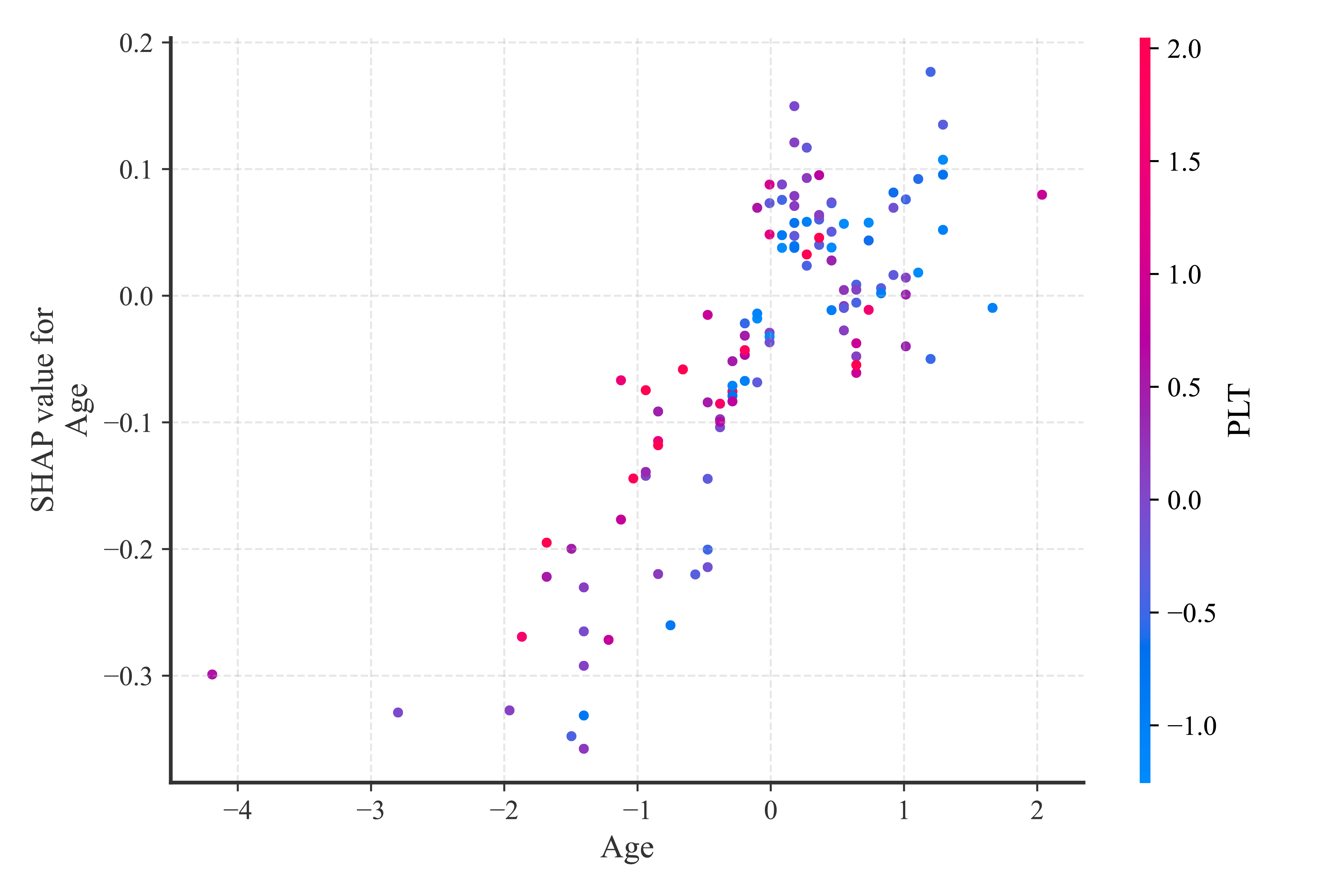

Supplement: Supplementary file 20 [file Image12.png]

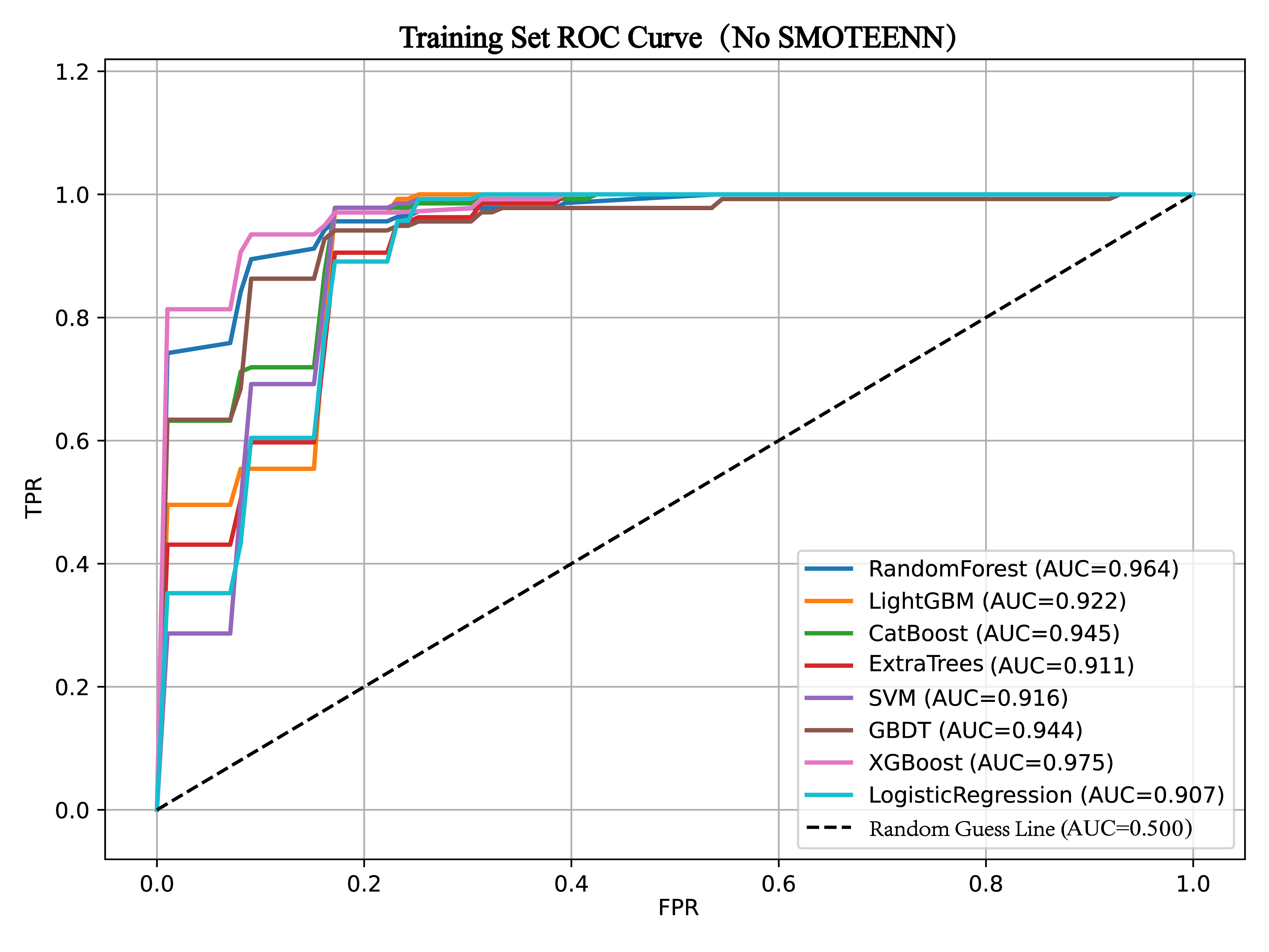

Supplement: Supplementary file 21 [file Image13.png]
